# Vision–language foundation models for multimodal medical image understanding and report generation: a narrative review with quantitative synthesis

**DOI:** 10.3389/fmed.2026.1958283

**Published:** 2026-10-26

**Authors:** Shahid Mehmood, Farman Matloob Khan, Abeer A. K. Alharbi, Abdul Khader Jilani Saudagar, Mona Alkhattabi, Muhammad Adnan Khan, Muhammad Amir Khan

**Affiliations:** 1 Department of Computer Science, Bahria University, Lahore, Pakistan; 2 College of Pharmacy, University of Sharjah, Sharjah, United Arab Emirates; 3 Information Systems Department, College of Computer and Information Sciences, Imam Mohammad Ibn Saud Islamic University (IMSIU), Riyadh, Saudi Arabia; 4 Department of Software, Faculty of Artificial Intelligence and Software, Gachon University, Seongnam-si, Republic of Korea; 5 Faculty of Computer and Mathematical Sciences, Universiti Teknologi MARA, Shah Alam, Selangor, Malaysia

**Keywords:** clinical evaluation, dataset bias, hallucination, medical image understanding, multimodal learning, radiology report generation, reporting standards, vision–language foundation models

## Abstract

Vision-language foundation models (VLFMs) for medical image understanding have advanced rapidly, yet evidence supporting clinical deployment remains fragmented. This narrative review synthesizes architectures, training paradigms, benchmarks, public datasets, and translation barriers. Drawing on more than 50 original studies published between 2020 and 2025, we provide a structured descriptive comparison rather than a statistical meta-analysis. In a focused audit of 13 representative studies, BLEU/ROUGE was reported by 31% (4/13), RadGraph F1 by 0% (0/13), CheXpert- or CheXbert-derived label evaluation by 46% (6/13), BERTScore by 8% (1/13), human expert evaluation by 31% (4/13), and inference latency or throughput by 0% (0/13). Because datasets, splits, candidate pools, and metrics differ, cross-task results are presented as exploratory benchmark indicators and not as a clinical-readiness scale. The synthesis identifies persistent concerns involving hallucination, limited interpretability, distribution shift, and narrow demographic and geographic coverage. We propose a minimum clinical validation and reporting standard aligned with the distinct purposes of FDA/Health Canada/MHRA and IMDRF Good Machine Learning Practice, the EU AI Act, TRIPOD+AI, and CLAIM, and identify eleven priority research directions. Medical VLFMs are promising, but progress should be judged by source-traceable, clinically grounded, and externally validated evidence.

## 1 Introduction

A typical academic radiology department generates thousands of imaging studies each week, each requiring a trained specialist to translate complex visual findings into a structured diagnostic report. Vision–language foundation models (VLFMs)—AI systems trained to jointly process medical images and natural language—may support report drafting, image-based question answering, retrieval, and triage. Since 2020, the field has expanded from contrastive dual encoders (ConVIRT, GLoRIA, MedCLIP) and unified multimodal encoders (MedViLL, MMBERT) to autoregressive report generators (R2Gen, UniXGen), multimodal LLM adapters (LLaVA-Med, Med-Flamingo, XrayGPT), and text-conditioned image generators such as RoentGen (1–3). These systems serve different tasks and should not be treated as directly interchangeable.

This review therefore provides a structured descriptive comparison of performance evidence rather than a pooled meta-analysis. Results are retained on their native task-specific scales, and values from different datasets or protocols are not combined statistically. The comparison suggests that discriminative tasks such as retrieval and classification generally have more mature benchmark evidence than open-ended generation; however, benchmark scores alone do not establish clinical utility, safety, or regulatory readiness. Report generation remains particularly sensitive to hallucinated findings, omissions, and protocol-dependent evaluation.

The evaluation audit also reveals uneven reporting. In a source-level audit of 13 representative studies (Table 1), 4/13 (31%) reported BLEU or ROUGE, 0/13 (0%) reported RadGraph F1, 6/13 (46%) used CheXpert- or CheXbert-derived label evaluation, 1/13 (8%) reported BERTScore, 4/13 (31%) included human expert evaluation, and 0/13 (0%) reported inference latency or throughput. RoentGen's domain-expert image assessment is counted as human evaluation but its image-generation metrics are not coded as report metrics; LLaVA-Med's GPT-4 proxy assessment is not counted as human evaluation, whereas Med-Flamingo's BERT-similarity score, defined as the BERTScore F1, is counted as BERTScore. These proportions describe only the displayed audit sample and should not be interpreted as field-wide prevalence estimates.

**Table 1:** Evaluation-reporting coverage in the focused 13-study audit. N/A identifies a metric family that is not applicable to the study's primary task; percentages retain the fixed denominator *n* = 13 and are not field-wide prevalence estimates.

Model (Year)	Dataset(s) Tested	BLEU/ROUGE	RadGraph F1	CheXpert Labels	BERTScore	Human Evaluation	Inference Latency/Throughput Reported
R2Gen (2020) (4)	MIMIC-CXR, IU X-Ray	✓	✗	✓	✗	✗	✗
GLoRIA (2021) (5)	MIMIC-CXR	✗	✗	✓	✗	✗	✗
ConVIRT (2022) (6)	MIMIC-CXR	✗	✗	✓	✗	✗	✗
MedCLIP (2022) (7)	MIMIC-CXR	✗	✗	✓	✗	✗	✗
RoentGen (2022) (8)	MIMIC-CXR	N/A (image generation)	N/A	N/A	N/A	✓	✗
MMBERT (2021) (9)	VQA-RAD	N/A (VQA accuracy)	✗	✗	✗	✗	✗
MedViLL (2022) (10)	MIMIC-CXR, VQA-RAD	✓	✗	✓	✗	✗	✗
BioViL-T (2023) (11)	MIMIC-CXR (current and prior images); MS-CXR-T	✓	✗	✓	✗	✗	✗
BiomedCLIP (2023) (12)	PMC-15M pretraining; ROCO retrieval	N/A (retrieval)	✗	✗	✗	✗	✗
LLaVA-Med (2023) (13)	PMC-15M-derived instruction data; biomedical VQA	N/A (VQA)	✗	✗	✗	✗	✗
Med-Flamingo (2023) (3)	VQA-RAD, PathVQA, Visual USMLE	✗	✗	✗	✓	✓	✗
XrayGPT (2023) (14)	MIMIC-CXR	✓	✗	✗	✗	✓	✗
PathChat (2024) (15)	PathVQA	N/A (VQA accuracy)	✗	✗	✗	✓	✗
**Percentage of Studies (*n*** **=** **13)**	**—**	**31% (4/13)**	**0% (0/13)**	**46% (6/13)**	**8% (1/13)**	**31% (4/13)**	**0% (0/13)**

Audit coding definitions: BLEU/ROUGE denotes lexical-overlap reporting for generated text; RadGraph F1 denotes graph-based entity/relation scoring; CheXpert labels denotes evaluation against labels produced by the CheXpert or CheXbert labeler, either as image-classification targets or as label agreement of generated reports; BERTScore denotes embedding-based semantic similarity, including Med-Flamingo's BERT-similarity score (BERTScore F1); human evaluation requires a human reader or domain expert rather than GPT-4 or another automated proxy; and inference reporting requires latency or throughput. RoentGen's expert image assessment is counted only as human evaluation, while its image-generation metrics are N/A to report generation.

Denominator note: All percentages use the 13 displayed studies. N/A entries identify task-inapplicable metrics and are not reclassified as positive reports. The audit is descriptive of this sample, not a field-wide prevalence estimate.

Beyond evaluation methodology, four systemic barriers constrain translation to clinical practice. First, generative VLFMs can hallucinate findings, producing clinically plausible but factually absent statements that surface-level language metrics may miss. Second, interpretability remains insufficient: most systems cannot reliably ground textual assertions in specific image regions. Attention maps or generated rationales can be useful candidate interfaces, but they do not by themselves demonstrate causal or faithful model reasoning and require explicit validation (16, 17). Third, robustness under distribution shift is poorly tested; models trained on one institution's imaging protocol may degrade at another. Fourth, the evidence base remains geographically and demographically narrow because many influential evaluations rely on single-institution chest-radiography resources such as MIMIC-CXR, with limited representation of pediatric, non-English-speaking, and low- and middle-income-country populations. Because this narrative review did not enumerate every benchmark in the field, no field-wide percentage is assigned to this limitation. Models validated only on a narrow database carry unresolved generalizability risks for the populations they are intended to serve.

Several high-quality reviews have surveyed related territory: Azad et al. examined medical foundation models broadly; Zhang and Metaxas surveyed radiology report generation and VQA methodology; and Hartsock and Rasool mapped medical VLMs (1, 18, 19). This review complements those contributions through five focused elements: (1) a source-traceable, cross-task descriptive performance comparison; (2) an evaluation-method audit with explicit denominators; (3) a critical appraisal of public-dataset limitations; (4) a synthesis of evidence on hallucination, interpretability, and distribution-shift fragility; and (5) a minimum clinical validation and reporting checklist that distinguishes reporting guidance, lifecycle practice, and legal obligations.

The paper is organized as follows. Section 2 defines the scope of multimodal medical AI and the task landscape. Section 3 presents a taxonomy of VLFM architectures with representative systems. Section 4 describes training paradigms and their trade-offs. Section 5 explains the narrative-review approach and presents benchmarks, evaluation metrics, and a structured quantitative synthesis. Section 6 critically appraises public datasets. Section 7 analyzes barriers to clinical translation. Section 8 maps eleven priority future research directions. Section 9 presents the proposed minimum clinical validation and reporting standard.

## 2 Background and scope

Multimodal medical image understanding refers to AI systems that jointly interpret medical images (e.g., radiographs, CT and MRI scans, pathology slides, and ultrasound) and accompanying text such as radiology reports, clinical notes, or questions (20–25). This reflects routine clinical practice: physicians combine visual findings with patient history and prior reports to reach diagnoses. VLFMs attempt to replicate this capability by learning from large multimodal datasets. In the medical context, a VLM typically consists of an image analysis module and a language module that are trained together on paired image-text data. During training, the model may encounter a chest x-ray and its corresponding radiology report, a pathology image along with the pathologist's description, or an MRI and its accompanying clinical note. By ingesting these pairs, the model learns aligned representations of visual and textual features, building a joint understanding of medical concepts in both modalities.

Foundation models are generally characterized by broad pretraining, scale, reuse, and adaptation capacity across downstream settings (26–28). For this review, a vision-language model (VLM) is any model that jointly processes visual and textual information, whereas a vision-language foundation model (VLFM) is a VLM pretrained on substantial and sufficiently broad image-text data for reuse or adaptation across more than one task, dataset, or clinical setting. We use three operational scope labels: task-specific (TS), optimized for a narrow task or protocol; domain foundation (DF), broadly pretrained within a biomedical or medical-imaging domain and reusable across tasks; and generalist foundation (GF), broadly pretrained across general-domain data and adaptable across domains and tasks. These labels describe the evidence available in the cited work, not a universal threshold. Classic report generators and narrow image-synthesis systems are retained only as explicit task-specific comparators.

Key vision–language tasks in the medical domain include: Radiology Report Generation (RRG) automatically producing the diagnostic report for a medical image; Visual Question Answering (VQA) answering natural language questions about medical images; Captioning and Description generating accurate captions for images (e.g., describing a pathology slide); Cross-modal Retrieval finding the matching report given an image or vice versa; and Image–Text Classification, e.g., predicting disease labels from an image using auxiliary text data. Among these, radiology report generation and medical VQA have received particular attention as challenging benchmarks for multimodal understanding. Report generation demands not only identifying findings in an image but also communicating them in coherent, clinically accurate language, effectively requiring both vision recognition and language generation skills (29–31). Medical VQA, on the other hand, tests a model's ability to reason about an image to answer arbitrary questions, which may involve fine-grained image analysis or reference to medical knowledge (32).

The scope of this review is defined as follows. We focus on models that take medical images and textual inputs, producing textual outputs (or aligned image-text embeddings) as part of a unified vision–language modeling approach. We therefore cover models for report generation, image–text retrieval, and VQA. We do not cover pure vision models (e.g., segmentation networks without any text component) or pure language models (text-only models for clinical notes) except in how they contribute to or integrate with multimodal systems. We also emphasize works from roughly 2020 onwards, corresponding to the surge of interest in “foundation” VLMs in medicine. Earlier works on radiology report generation using CNN-LSTM or template-based methods are discussed for context but are not the primary focus, because recent surveys have covered those techniques (33, 34). Instead, we concentrate on models that use transformers, large-scale pretraining, and multimodal fusion.

To ground the discussion, consider an example scenario within our scope: an AI assistant processes a patient's chest x-ray and possibly some clinical context (such as age and symptoms) to automatically draft a radiology report. This involves visual analysis (detecting opacities, fractures, etc.) and language generation (forming sentences about those findings). A vision–language foundation model for this task might be pre-trained on hundreds of thousands of image-report pairs (such as the MIMIC-CXR dataset), allowing it to learn the typical patterns of abnormalities and how they are described in text (1). After pretraining, the model can be fine-tuned to ensure the generated reports are fluent and medically accurate. Other modalities (such as an ophthalmology VLM, which explains retinal images) or interactive tasks (such as answering a question about a medical image) can be adapted to similar foundation models. In each case, the common aim is to train a model that represents both images and text and can map between them in a clinical context.

In summary, this review covers multimodal medical AI systems that integrate visual and textual information. Its scope includes several imaging modalities (radiography, cross-sectional imaging, pathology, etc.) and the variety of texts (reports, captions, questions, knowledge bases) with an emphasis on foundation models trained at scale on these data. In the following sections, we first introduce the state-of-the-art architectures enabling such models (Section 3) and then describe their training regimes (Section 4), evaluation methodologies (Section 5), data resources (Section 6), and the broader challenges and future trends (Sections 7, 8) in developing trustworthy vision–language AI for healthcare. Throughout, we cite representative and influential works from 2020 to 2025 to illustrate key points and to show how the field has evolved over this period.

## 3 Representative model architectures

Vision-language models in the medical domain can be categorized by architecture, information flow, and scope. Figures 1–4 summarize dual-encoder contrastive systems, unified fusion encoders, autoregressive image-to-text generators, and vision-to-language bridges for multimodal large language models. Table 2 separately identifies task-specific, domain-foundation, and generalist-foundation examples so that architecture class is not treated as synonymous with foundation-model status.

**Figure 1:**
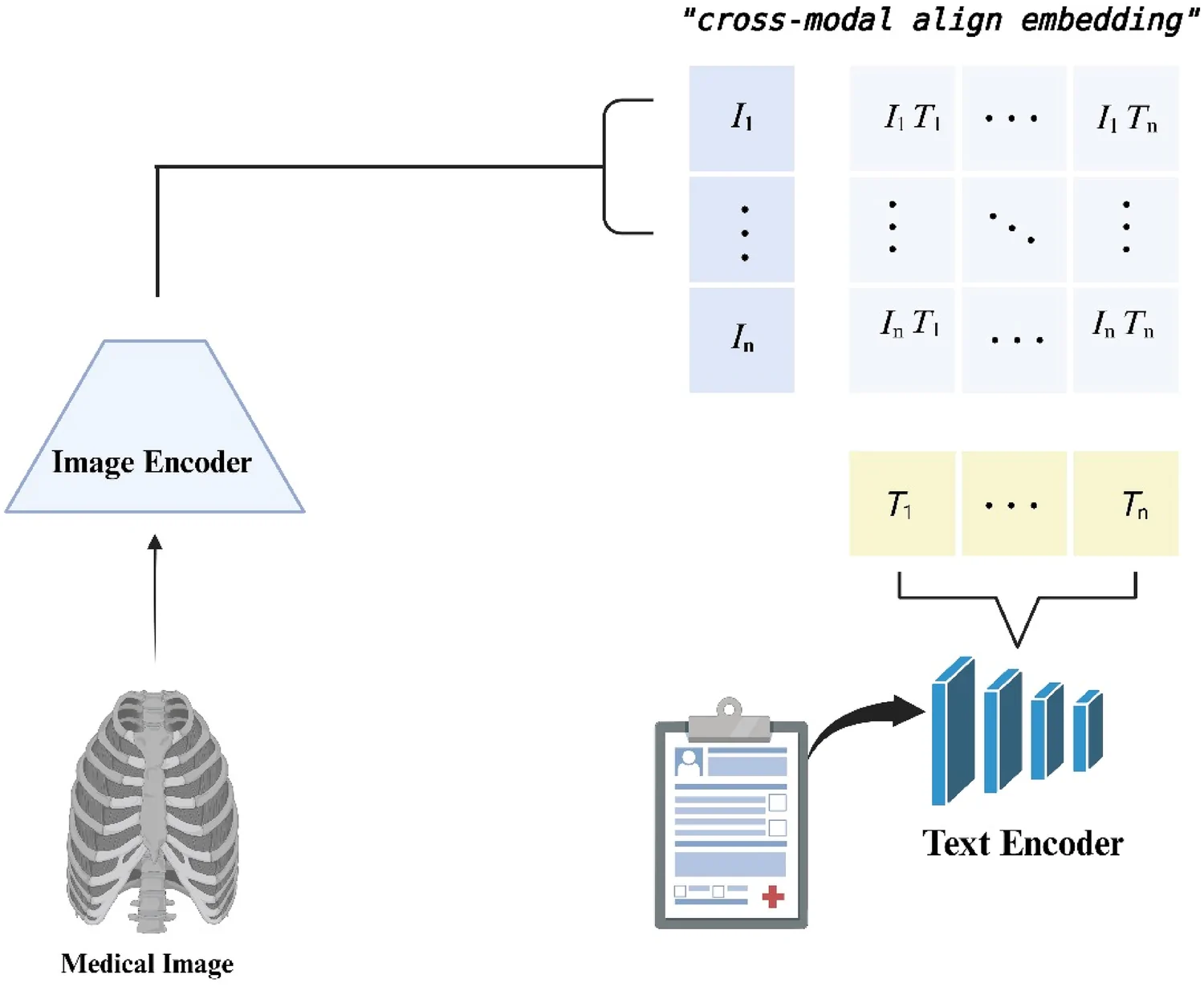
Dual-encoder contrastive architecture. A convolutional neural network (CNN) or vision transformer (ViT) encodes the medical image, a transformer encodes the associated text, and a contrastive objective aligns paired embeddings in a shared space for retrieval or zero-shot transfer.

**Figure 2:**
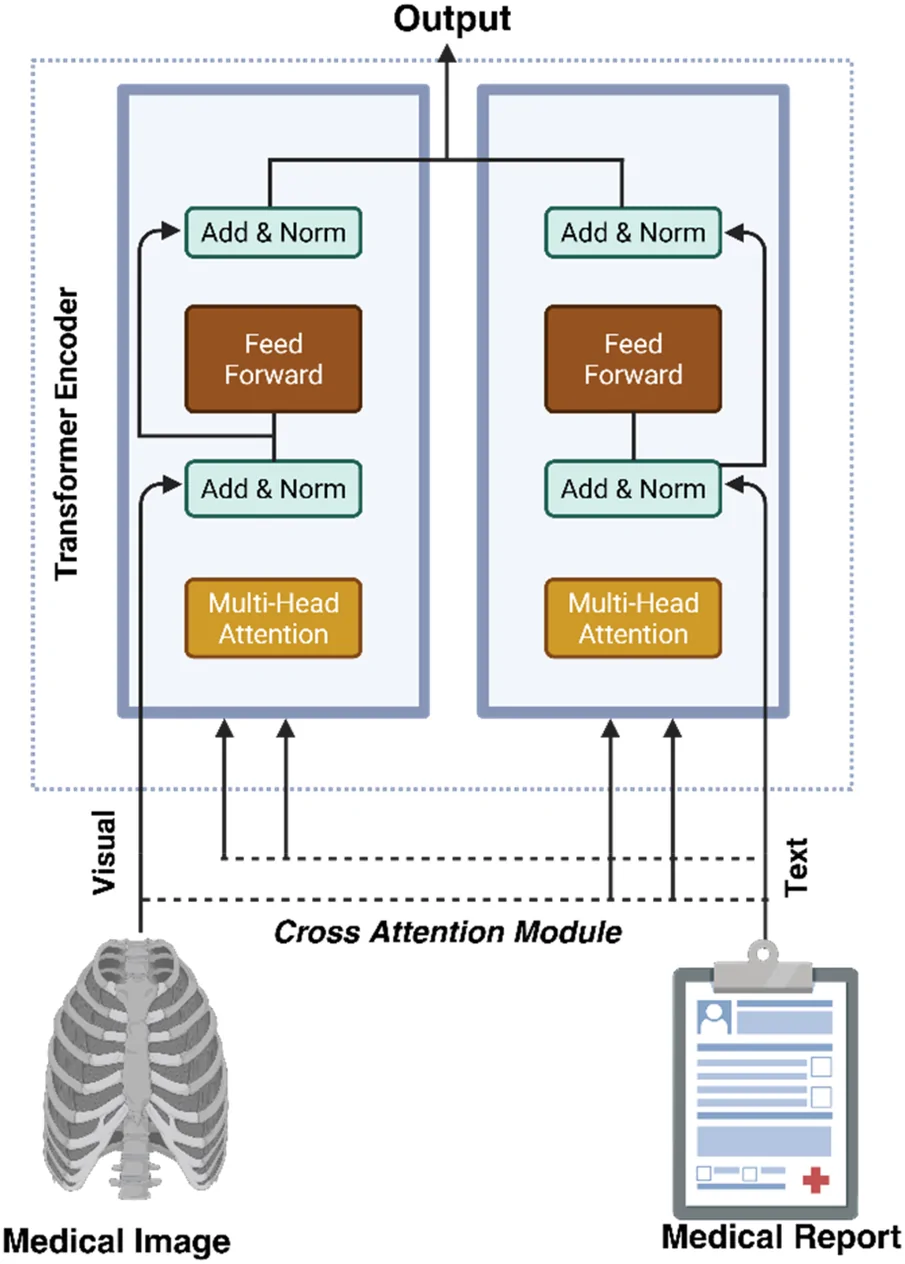
Unified or fusion encoder. Image patches or region features and text tokens are jointly processed by a multimodal transformer, enabling bidirectional cross-modal interaction before a task-specific output head.

**Figure 3:**
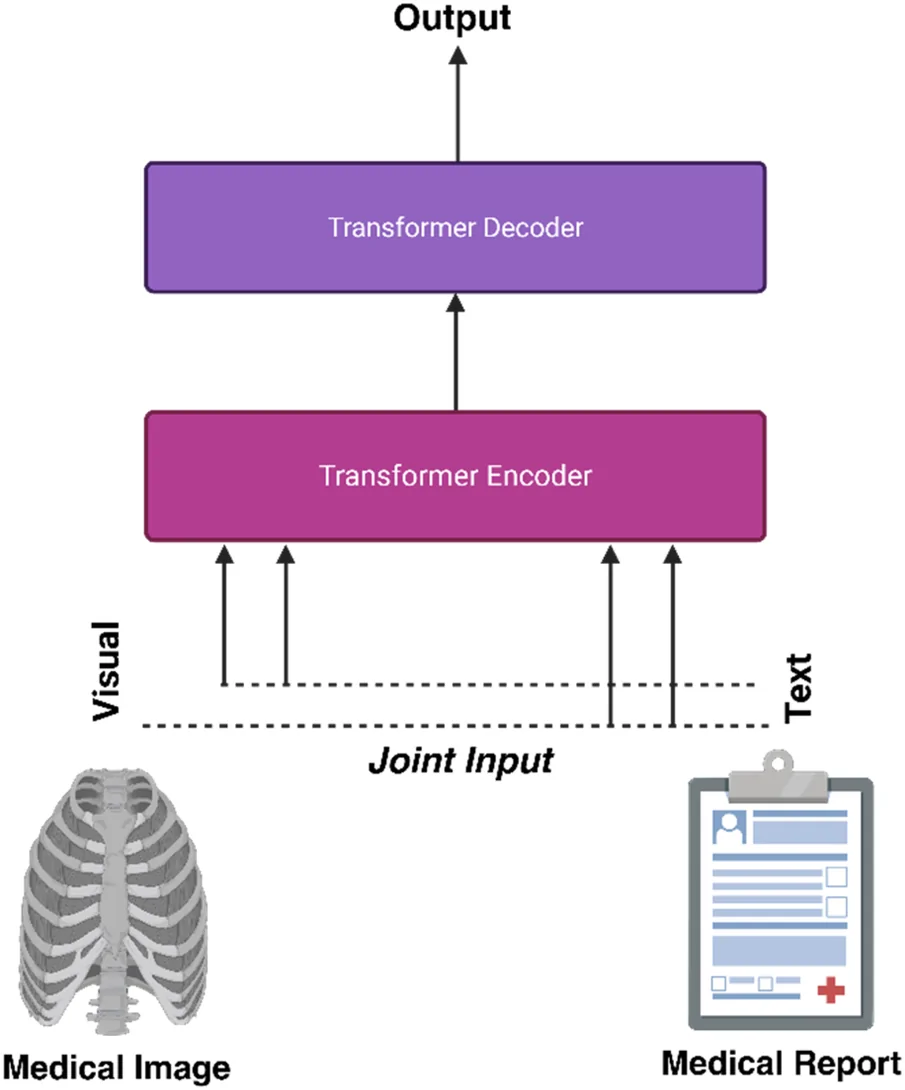
Autoregressive image-to-text encoder-decoder. A CNN or ViT encodes the image; a transformer decoder attends to visual features and predicts report tokens sequentially.

**Figure 4:**
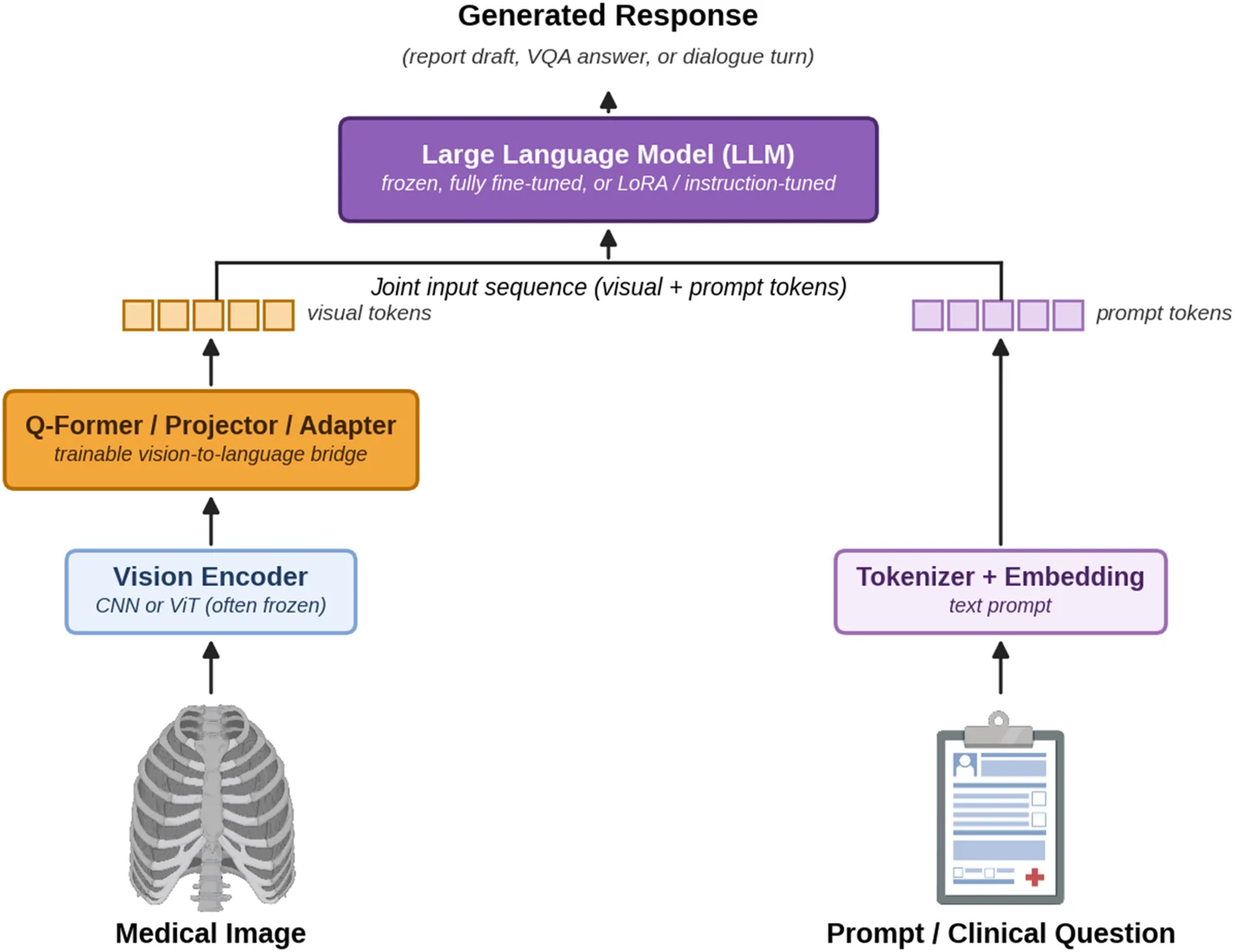
Adapter-based multimodal large language model (LLM). A vision encoder extracts image features, which a Q-Former, linear projector, or adapter maps to visual tokens; these tokens are combined with the embedded text prompt and passed to the LLM, which generates the response (e.g., a report draft or a VQA answer).

**Table 2:** Architecture taxonomy and operational scope of representative vision-language models. TS, task-specific; DF, domain foundation; GF, generalist foundation. Scope labels reflect breadth of pretraining and documented reuse, not clinical readiness.

Model Type	Representative Models	Core Mechanism	Strengths	Limitations	Evidence Maturity
Dual encoder	CLIP (GF); ConVIRT (DF); GLoRIA (DF); ALIGN (GF); MedCLIP (DF)	Separate image and text encoders aligned contrastively	Scalable retrieval and zero-shot transfer	Limited token-level cross-modal reasoning	Research benchmark
Fusion encoder	MedViLL (DF); MMBERT (DF)	Image and text tokens interact within a shared transformer	Fine-grained multimodal fusion	Compute-intensive; task-specific evidence	Research benchmark
Vision-LLM bridge	BLIP-2 (GF); LLaVA-Med (DF); Med-Flamingo (DF); XrayGPT (DF)	A vision encoder connects to an LLM through a Q-Former, projector, or adapter	Flexible prompting and dialogue	Hallucination, calibration, and grounding concerns	Emerging evidence
Report generator	R2Gen (TS); UniXGen (TS)	Visual tokens condition sequential report generation	Task-focused report generation	Sensitive to templates and factual errors	Benchmark; limited prospective evidence
Image generator	RoentGen (TS)	Latent diffusion generates chest x-ray images from text	Image synthesis and augmentation	Not a radiology report generator	Research prototype

### 3.1 Dual-encoder contrastive models (image–text alignment)

The dual-encoder architecture consists of two separate networks: one for images and one for text. Each takes its respective input (e.g., an image and its corresponding report) and produces a feature embedding. The model is trained so that embeddings of matching image–text pairs are pulled together, while non-matching pairs are pushed apart in the shared embedding space. This is typically achieved with a contrastive learning objective (often InfoNCE loss), which encourages correct image–caption pairings to have higher similarity scores than mismatched pairings. The seminal example of this approach is CLIP (Contrastive Language–Image Pretraining) by (35) which showed that contrastively trained vision and text encoders can achieve strong zero-shot classification and retrieval performance by aligning multimodal features. In the medical domain, ConVIRT (Contrastive Visual Representation Learning from Text) was one of the first to apply this idea to radiology images and reports (6), using chest x-rays and paired text to learn image representations without any manual labels. Subsequent medical models followed suit with improved alignment strategies; for instance, GLoRIA (5) introduced a global-local contrastive loss to better align fine-grained image regions with report sentences, boosting the localization of findings in chest x-rays. These dual-encoder models do not generate text; instead, they are suited to image–text matching tasks: given a chest x-ray and a pool of report sentences, a contrastive model can identify which sentences are likely to describe the image (36, 37). This makes them useful for retrieval-based diagnosis (finding similar past cases/reports for a given image) and as a pretraining backbone; the image encoder can be reused for downstream tasks (yielding strong initialization for classification or detection models) (38).

A key advantage of dual-encoders is scalability. Because the image and text towers are separate, large datasets of paired but weakly labeled data can be utilized, e.g., any image with an associated report, even if the report contains more information than just a caption (39). Medical datasets, such as MIMIC-CXR (377,000 chest x-rays and reports) and PadChest (160,000 images with Spanish/English labels), naturally provide paired data (40, 41). Models such as BioViL (42) and MedCLIP (7) leverage contrastive pretraining on these large corpora to produce image embeddings that are aligned with the medical text domain, enabling zero-shot recognition of pathologies. For example, BioViL pairs a ResNet-50 image encoder with a radiology-specific text encoder (CXR-BERT), was trained on MIMIC-CXR chest radiograph–report pairs, and was evaluated on zero-shot and fine-tuned classification, segmentation, and phrase grounding; its authors also released MS-CXR, a radiologist-verified phrase-grounding benchmark (42). Another example is BiomedCLIP (12), which scaled up contrastive learning to 15 million figure-caption pairs from PubMed articles, producing a general biomedical image–text model that can handle radiology images as well as photos or diagrams. In ophthalmology, RET-CLIP applied the approach to retinal fundus photographs paired with diagnostic reports (43), whereas Mammo-CLIP (44) did so for mammography exams; both studies reported zero-shot classification of abnormalities using the learned image embeddings.

Overall, dual-encoder VLMs have become a popular foundation in medical imaging because they are simple, computationally efficient, and effective for representation learning. Their aligned embeddings can be used directly for multi-label image classification (via a nearest-neighbor approach or a linear probe) and for image–report retrieval (finding the closest matching report in a database for a given image, which itself can assist clinicians). However, a limitation is that these models do not inherently generate text. Thus, they are less suited for tasks that require detailed free-text output, such as whole report generation. Also, by design, dual encoders treat image and text separately until a final similarity comparison, which means they may miss some granular interactions between modalities (e.g., linking a specific phrase to an exact location on the image).

While their scalability and simplicity make dual-encoders highly attractive for pretraining, they remain limited in clinical reasoning capability. By separating visual and textual processing until the embedding comparison stage, they often fail to model fine-grained semantic alignments, for example, associating the phrase “right upper lobe opacity” with its exact spatial region. Consequently, while dual-encoders provide strong zero-shot classification and retrieval performance, they underperform in compositional reasoning or report generation, where detailed cross-modal understanding is critical. Nevertheless, many recent medical VLM studies use contrastive dual-encoder pretraining as a first stage because it is stable and scales well.

### 3.2 Unified multimodal encoders (single-stream attention)

Unified multimodal encoder models take a different approach. Instead of having separate networks for each modality, they feed both image and text data into a single transformer model, allowing for joint processing. Typically, the image is first decomposed into a series of region features or patch embeddings (for example, using a Vision Transformer or a CNN backbone), and text is tokenized into embeddings. These sequences are concatenated or combined and passed through transformer layers that perform self-attention over the entire multimodal sequence (45). This architecture is sometimes called a single-stream or fusion encoder, as exemplified by models like VisualBERT (46) and LXMERT (47) in the general domain (though those earlier models had slight differences in how they fused inputs). The key idea is that at each attention layer, cross-modal interactions can occur: the model can attend to an image region based on a word or vice versa, learning fine-grained relationships between visual and textual elements.

In the medical setting, unified encoders are suited to tasks that require joint reasoning over image and text. One notable example is MedViLL (10), which uses a single-stream transformer to combine a chest x-ray image (processed into patch embeddings via a CNN) with the text of a radiology report, and is trained with a combination of language modeling and alignment objectives. MedViLL demonstrated that this approach can improve performance on tasks such as predicting diagnostic labels with justification. By jointly attending to image findings and report text, the model better matches clinical findings with the visual evidence. Another example is MMBERT (9) (VisualBERT adaptation for radiology), which fuses images and text and showed gains in medical VQA by reasoning across modalities. Unified encoders are generally well suited to VQA tasks (where the question text and image must be considered together) and in tasks like report–image consistency checking (did a given report truly correspond to the image?). These models inherently capture interactions such as “the word ‘nodule’ aligns with this spot in the lung field” through their attention mechanisms.

The benefits of multimodal encoders come at a cost: they are computationally heavier, since the transformer now processes the combined length of image patches and text tokens. For large images or long reports, this can be memory-intensive. Moreover, training such models from scratch typically requires substantial paired data and is therefore less common than using dual encoders. Nevertheless, the approach remains attractive for tasks that need fine-grained cross-modal interaction.

In summary, single-stream transformer VLMs offer richer multimodal feature fusion than dual encoders, making them particularly well-suited for nuanced reasoning tasks in medicine (46). They have demonstrated strong results on benchmark datasets like VQA-Med (answering questions from radiology images) and have been applied to tasks such as matching patient clinical notes with imaging findings (to flag discrepancies or enrich reports). The trade-off is that they often require more training data or careful initialization (e.g., starting from a model like PubMedBERT for text and ImageNet for image) to converge. Some recent works attempt to mitigate data needs by pretraining unified models on non-medical image-text data and then fine-tuning on medical data. For example, starting from a COCO-pretrained ViLT (Vision-and-Language Transformer) and then fine-tuning on radiology data has been explored. When pre-trained on general-domain data and fine-tuned on small medical corpora, unified encoders often exhibit catastrophic forgetting or hallucination, indicating that domain transfer remains a non-trivial task. Nonetheless, as of 2025, fully unified models remain less common in medical AI than dual-encoder or encoder–decoder models, largely because of their complexity. This may change as larger multimodal medical datasets (and compute resources) become available, since unified encoders align well with the goal of holistic image-text understanding.

### 3.3 Encoder–decoder generative models (image-to-text generation)

Encoder–decoder models extend the multimodal pipeline by including a text generation decoder that produces free-text outputs conditioned on image features (and optionally input text). In these architectures, the image is first processed by an encoder (which could be a CNN, ViT, or even a multimodal encoder as above) to obtain a latent representation. Then, a decoder network, often a transformer decoder, generates a sequence of words (such as a report or caption) based on that representation. The decoder employs attention mechanisms to attend to the encoded visual features as it generates each word, ensuring the output text is grounded in the image content. Because these models are generative, they are a natural choice for report generation.

Radiology report generation has been a research topic for many years, and earlier encoder–decoder implementations used convolutional encoders with RNN (LSTM) decoders. Modern approaches, however, use transformer-based encoders and decoders for greater capacity and fluency. A representative model is R2Gen (4), which uses a CNN image encoder and a memory-driven transformer decoder and outperformed earlier RNN-based baselines on MIMIC-CXR language-generation metrics and CheXpert-labeler clinical-efficacy scores. RoentGen (8), which is sometimes grouped with these systems, belongs to a different class: it adapts a latent-diffusion (Stable Diffusion) model to synthesize chest radiographs from free-text radiology prompts, and its outputs were assessed with image-quality measures and by domain experts rather than with report-generation metrics. RoentGen is therefore a text-conditioned chest x-ray image-generation model, not a report-generation encoder–decoder; it is included in this review only as a comparator (Tables 2–4 and Section 8.5).

**Table 3:** Principal training objectives used in medical vision-language systems. Representative examples are assigned to their primary objective; individual systems may combine several objectives.

Training Paradigm	Typical Objective	Representative Models	Advantages	Drawbacks	Suitable Use-Cases
Image–text contrastive learning	Align paired image and text embeddings	CLIP, ConVIRT, GLoRIA, MedCLIP	Retrieval and zero-shot transfer	Needs large, well-curated pairs	Retrieval, classification, tagging
Masked language/multimodal modeling	Recover masked tokens using image–text context	MedViLL, MMBERT	Learns fused representations	Can remain text-centric	VQA, retrieval, representation learning
Masked visual modeling	Recover masked visual tokens or features	M3AE and medical masked-autoencoder variants	Encourages visual context modeling	May emphasize low-level reconstruction	Representation pretraining
Instruction tuning	Follow multimodal natural-language instructions	LLaVA-Med, Med-Flamingo, XrayGPT	Flexible interaction	Requires curated instructions and safety evaluation	VQA and clinical-assistant research
Autoregressive generation	Predict report or answer tokens from image features	R2Gen, UniXGen	Direct task optimization	Dataset bias and hallucination	Report generation and multimodal generation
Text-conditioned diffusion	Denoise latent visual representations conditioned on text	RoentGen	Realistic image synthesis	Image-generation evaluation differs from report evaluation	Synthetic chest x-ray generation
Region-phrase grounding	Align local image regions with report phrases or clinical entities	GLoRIA, BioViL	Supports localization and fine-grained alignment	Alignment is not necessarily causal or faithful explanation	Localization, retrieval, weakly supervised grounding

**Table 4:** Scope and source audit of representative chest-radiology vision–language systems.

Model (Year)	Primary Task/Architecture	Dataset/Protocol	Reported Quantitative Result	Source Note
R2Gen (2020) (4)	Report generation; memory-driven encoder–decoder	MIMIC-CXR; primary-paper protocol	BLEU-1 0.353; BLEU-4 0.103; METEOR 0.142; ROUGE-L 0.277; CheXpert-labeler clinical-efficacy F1 0.276; RadGraph NR	Values retained from the cited primary study
RoentGen (2022) (8)	Text-to-image latent diffusion	MIMIC-CXR	N/A for report generation	Generates chest x-ray images; not a report generator
GLoRIA (2021) (5)	Contrastive representation learning	MIMIC-CXR	N/A for autoregressive report generation	Primary study emphasizes representation learning
BioViL-T (2023) (11)	Temporal (current + prior image) vision–language pretraining; CXR-BERT-initialized text model used as decoder	MIMIC-CXR v2 with prior images; primary-paper protocol	BLEU-4 9.2; ROUGE-L 29.6; CheXbert 31.7; TEM 17.5 (0–100 scale)	Values retained from the cited primary study; test split differs from R2Gen. The original BioViL (42) did not evaluate report generation
LLaVA-Med (2023) (13)	Biomedical conversation and VQA	PMC figures and biomedical VQA	N/A for MIMIC-CXR report generation	Primary paper does not report MIMIC-CXR BLEU or RadGraph
CXR-RePaiR (2024) (48)	Retrieval-based report generation	Study-specific retrieval protocol	NR in this audit	Consult the primary paper; not ranked across incompatible protocols
XrayGPT (2023) (14)	Conversational CXR summarization	MIMIC-CXR with instruction data	NR in this audit	No standardized value attributed without a matching protocol
Human expert studies (49, 50)	Expert assessment	Protocol dependent	Study-specific	No universal RadGraph or CheXpert human ceiling assumed

One challenge with encoder–decoder models is that they typically require fully paired data (images with complete reports) for training. Such datasets, although available (e.g., MIMIC-CXR), are smaller in scale compared to image-caption pairs used in contrastive learning. Writing a detailed report also demands capturing subtle findings and their clinical significance, which models struggle to learn when training data are limited or when radiologists’ phrasing varies widely. As a result, pure encoder–decoder VLMs were less common in early foundation model literature, but they are gaining traction as researchers combine them with large-scale pretraining. For example, BioViL-T extends BioViL with a hybrid CNN–transformer multi-image encoder that also processes the prior chest radiograph when available; for report generation, its CXR-BERT-initialized text model cross-attends to the image features, with causal attention between text tokens, so that one pretrained backbone supports both alignment and generation (11).

Importantly, encoder–decoder frameworks allow for incorporating conditional inputs beyond just the image. One can feed patient metadata or a short text prompt into the encoder along with the image. For instance, a model might take an image, along with the text “Indication: cough and fever,” as input and produce a tailored report that addresses that specific clinical context. This flexibility is a strength of encoder–decoder designs and aligns with clinical usage, where reports are influenced by the clinical question at hand. Models like PromptSRC (51) explore prompt-based conditioning, where a text prompt guides the generation of reports (e.g., focusing on specific findings).

As shown in Figure 3, encoder–decoder integration typically means the image and text (if any) are encoded jointly, and then the decoder generates the output text. Some architectures also allow iterative generation, for example, generating one sentence at a time and feeding it back in; however, most current models generate in a single pass. In a related variant, shown in Figure 4, the decoder is a large language model adapted to accept visual input; this leads to the instruction-tuned multimodal LLMs discussed in Section 3.4.

Encoder–decoder models have improved automated report drafting, but important errors remain. Common issues include omitted findings (a pathology present in the image is not reported) and hallucinated findings (a finding is reported that is not present in the image), often reflecting biases in the training reports (49). Furthermore, evaluation of report generation frequently relies on BLEU or ROUGE, which correlate poorly with clinical correctness. The absence of standardized benchmarks complicates cross-study comparison and slows regulatory validation. Some works have started to address these issues by augmenting generation with constraints or post-validation. For instance, the X-REM framework (48) retrieves similar past reports and uses them to guide the decoder, aiming to avoid out-of-distribution statements. Another approach is reinforcement learning: some studies have applied reinforcement learning with human feedback (or proxy rewards, such as CheXpert accuracy) to adjust the decoder's outputs to be more factual. While not yet widespread, these strategies hint at how encoder–decoder VLMs are evolving to meet clinical reliability standards.

In summary, transformer-based encoder–decoder architectures underpin most current medical report-generation systems. They can produce human-readable narratives from images, a central goal for AI assistants in radiology (50). The current generation of models has made strides in fluency and relevance of generated reports, but ensuring complete clinical accuracy and appropriateness remains an active area of research (discussed further in Section 7 on Challenges). Next, we consider a subclass of encoder–decoder models that use general-purpose large language models for multimodal tasks.

### 3.4 Large instruction-tuned multimodal models

A more recent development is the emergence of instruction-tuned vision–language models, which combine large language models with vision encoders to support interactive use. These systems are designed to follow natural language instructions or engage in dialogue about images, essentially multimodal AI assistants for medical applications. They can be seen as encoder–decoder models, where the decoder is a pre-trained large language model (LLM), such as GPT-4, PaLM, or LLaMA, and the image information is injected via a suitable interface (e.g., a visual encoder whose outputs are fed into the LLM's context). By fine-tuning such a model on medical image–text instruction data (e.g., question-answer pairs, conversational radiology report corrections), we obtain a system that can converse with clinicians about images, explain its reasoning, or generate customized reports on demand.

Several examples appeared between 2023 and 2025. Med-Flamingo (3) adapts DeepMind's Flamingo VLM (which uses a few-shot prompting approach with images) to the medical domain. It was trained on a curated dataset of medical textbook images (MTB) and their corresponding descriptions, enabling it to perform few-shot learning on tasks such as describing an x-ray or answering questions about an anatomical diagram. LLaVA-Med (13) builds on the open-source LLaVA (Large Language and Vision Assistant) model, which connects a CLIP ViT image encoder to a LLaMA-based language model (52). LLaVA-Med was further trained on medical instruction-following data (such as instructions and responses generated from medical exam QA or clinician dialogues) to specialize it for healthcare. The result is a system that, given an image and a prompt such as “Describe any abnormalities and suggest a diagnosis,” can produce a paragraph-length answer; such answers still require clinical verification. Another model, Visual Med-Alpaca (53), extends the Alpaca family of instruction-tuned LLMs by adding a vision front-end. It was fine-tuned on combined image-question-answer triples from datasets such as VQA-RAD and SLAKE, with the aim of supporting both report-style generation and VQA within a single conversational model.

Domain-specific assistants include XrayGPT, which connects a medical image encoder to a language model for conversational chest-radiograph analysis, and PathChat, which combines a pathology foundation encoder with a pretrained language model and instruction tuning (14, 15). Their fluent responses and generated rationales should be treated as outputs to be verified, not as evidence that the model has exposed its internal reasoning.

Instruction-tuned systems can produce flexible, context-sensitive dialogue because the language model contributes broad linguistic knowledge. That fluency is useful for interaction but can conceal unsupported clinical inferences. For example, adding symptoms to an image prompt may produce a more specific narrative without proving that the image supports the diagnosis. Outputs should therefore separate observed image findings, supplied clinical context, retrieved evidence, and model inference.

Instruction-tuned multimodal models remain vulnerable to hallucination, prompt sensitivity, distribution shift, and overconfident language. Retrieval or tool use may improve grounding only when source quality, retrieval failure, and citation fidelity are evaluated. These systems are research prototypes rather than clinically authorized products and require domain-specific validation, privacy controls, calibrated uncertainty or abstention, and standardized human-AI interaction studies before clinical deployment.

Table 2 separates architecture from model scope. ALIGN and MedCLIP are dual encoders; BLIP-2 uses a Q-Former bridge between a frozen vision encoder and language model; LLaVA-Med uses a learned projection to a multimodal LLM; R2Gen is a task-specific report generator; and RoentGen is a text-conditioned latent-diffusion image generator. Evidence maturity remains distinct from deployment readiness.

## 4 Training paradigms

Vision-language foundation model training is a combination of multimodal large-scale pretraining and fine-tuning on individual tasks. In this section, we describe the popular training paradigms, such as self-supervised objectives applied in pretraining, transfer learning methods, and the fine-tuning or adapting methods (such as instruction tuning and prompt learning) that are used to specialize models on tasks such as report generation or VQA. While the underlying objectives resemble those used in general-domain AI, medical VLFMs face unique challenges: limited data scale, high annotation cost, and the need for factual correctness rather than mere fluency. Below, we critically examine major pretraining and fine-tuning strategies, highlighting their empirical benefits, trade-offs, and unresolved issues.

### 4.1 Multimodal pretraining objectives

A hallmark of foundation models is unsupervised or self-supervised pretraining on enormous datasets. For vision–language models, several pretraining objectives have proven effective:

#### 4.1.1 Image–text contrastive (ITC) learning

As used in CLIP and ConVIRT, this objective trains an image encoder and text encoder to produce similar embeddings for paired image-text data and dissimilar embeddings for mismatched pairs (35). The model essentially learns to understand which pieces of text could describe which image. In the medical domain, ITC is often the first stage, e.g., in ConVIRT, BioViL, and MedCLIP, which use contrastive objectives on chest radiograph–report data to initialize their encoders (6, 7, 42). However, its reliance on coarse pairwise similarity often results in semantic dilution; the model learns which image matches which report, but not why. Consequently, ITC yields discriminative features that transfer well to classification but lack fine-grained reasoning capacity. Future progress requires hybrid objectives coupling contrastive loss with localized supervision (e.g., region-phrase alignment or pathology-level grounding).

#### 4.1.2 Image–text matching (ITM)

A related task where the model (often a unified encoder) outputs a probability that a given image and text are a true pair. This is like a binary classification version of contrastive learning. Some medical VLMs (e.g., UNITER variants for radiology) utilize ITM to train a fusion encoder to distinguish between aligned and misaligned image-report pairs, thereby improving cross-modal alignment. Yet its performance depends heavily on the quality of negative sampling; naive random mismatches can make the task trivial. In medicine, where reports often share phrasing, “hard negative” selection (similar but clinically distinct cases) is essential but underexplored. Without it, models risk shortcut learning, memorizing surface lexical patterns rather than visual-semantic correspondences.

#### 4.1.3 Masked language modeling (MLM) with vision

Adapted from BERT's text-only MLM, this involves masking out some words in the text (e.g., specific medical terms) and training the model to predict them, while providing the corresponding image as context. The image essentially acts as additional context to fill in the blank. For example, if a report says “_____ in the right lung”, and the image shows a nodule, the model should infer “nodule” or “opacity”. Models like MedViLL employ MLM alongside vision, and more recent ones, such as MUMC (2023) (54), explicitly combine Masked Image Modeling and Masked Language Modeling losses. The MUMC model, for instance, masks random patches in the image and random tokens in the text, and trains a single transformer to reconstruct both, along with a contrastive loss, providing comprehensive multitask pretraining. Masked Language Modeling (MLM) faces several significant limitations when applied to vision tasks. Fundamentally, there's a mismatch between the design of MLM for discrete language tokens and the continuous, spatially structured nature of visual data. Additionally, vision transformers with MLM require substantial computational resources to process high-resolution images with numerous patches, and the masking-reconstruction process adds overhead compared to alternative self-supervised methods.

#### 4.1.4 Masked vision modeling (MVM)

The inverse of MLM hides parts of the image and uses the text (and remaining image) to predict them. Practically, this may involve the prediction of visual characteristics or labels of a masked area. Other methods are based on discretizing image patches (via a VQGAN codebook) and training image reconstruction as a language-modeling task. Although it is innovative, Masked Vision Modeling (MVM) has major drawbacks because the asymmetry between vision and language is inherent. The main point is that the process of reconstructing masked image patches using text is ambiguous and informationally sparse by its nature.

Moreover, MVM can also discretize vision into tokens, which makes images a representation of a symbolic sequence, but it may be optimizing to low-level visual reconstruction and not high-level semantic interpretation, which might not be able to capture the subtle meaning that language can. Consequently, MVM often provides a weaker and noisier training signal for vision-language alignment compared to objectives like contrastive learning. This is less common in medical VLMs but has been explored in cases such as reconstructing missing image slices from text descriptions.

#### 4.1.5 Image captioning (language modeling)

Another powerful paradigm is to train the model to directly generate text given an image, i.e., treating the entire radiology report as a target to be predicted autoregressively. This can be done by prefixing the image representation to a language model and training with next-word prediction (similar to how GPT is trained on text). SimVLM (45) did this by treating image patches as “pseudo-words” in a prefix to the text; some medical adaptations have followed suit (55, 56). Generative pretraining can teach the model the style and structure of reports; however, doing it from scratch requires a substantial amount of data. As a result, many systems rely on a hybrid: use contrastive or MLM pretraining for alignment, then a smaller amount of captioning-style training to tune the model for generation.

#### 4.1.6 Alignment with knowledge bases

A specialized objective for medicine is grounding the model in external knowledge. For example, a training task might have the model align an image with a text explanation or with clinical concepts. The knowledge [e.g., RadGraph labels or CheXpert labels) can be integrated through multitask training, which simultaneously predicts structured labels (such as 14 disease presence labels from CheXpert (57)] alongside report generation. This multitask learning can inject factual supervision and reduce unsupported statements in generated text. Yet, these approaches require high-quality, structured annotations, which remain limited outside of chest radiography. Furthermore, multitask training introduces optimization conflicts, and label supervision may over-constrain generation and reduce the flexibility of the output text.

### 4.2 Transfer learning and fine-tuning strategies

After broad pretraining, fine-tuning adapts a model to specific tasks or data distributions. In medical AI, transfer learning is crucial because distribution shifts (e.g., between hospitals or between modalities) can be significant. We highlight key paradigms:

- **Supervised Fine-Tuning (SFT):** The straightforward approach: take the pre-trained model and further train it on a labeled dataset for the target task (e.g., MIMIC-CXR report generation or VQA-RAD Q&A) (58). Almost all top-performing models do an SFT on the target task to specialize performance. For instance, a model pre-trained on MIMIC-CXR + OpenI might be fine-tuned on the IU X-Ray dataset for report generation to maximize BLEU/METEOR metrics. SFT is nearly universal but frequently overfits small datasets and erases pre-trained generalization (“catastrophic forgetting”). SFT usually involves a smaller learning rate and sometimes partial freezing of layers to avoid catastrophic forgetting of pre-trained knowledge. Comparative studies quantifying the extent to which pretraining knowledge persists after SFT are lacking, a significant omission for clinical reproducibility.
- **Domain-Adaptive Pretraining:** If a model is pre-trained on generic image-text data (say, natural images from the internet), an intermediate adaptive training on medical data (unlabeled) can be done. For example, using a general CLIP model and then continuing contrastive training on MIMIC-CXR for a few epochs to specialize it, effectively creating a MedCLIP. This has been shown to improve performance over using the generic model directly (7). Many medical VLMs initialize their weights from a non-medical foundation model (such as initializing the vision backbone from ImageNet or the text backbone from PubMedBERT) and then undergo domain-specific training (59). Yet this process can amplify existing biases if medical data are homogeneous (e.g., Western populations, limited device variety). Few papers test cross-site generalization; most report single-center results, which limit their external validity.
- **Parameter-Efficient Tuning:** Given the size of foundation models, methods such as prompt tuning and LoRA (Low-Rank Adaptation) are used to avoid fine-tuning all parameters (60). For instance, prefix tuning involves learning a set of virtual tokens (vectors) that are prepended to the input and are trained to steer the model for a new task (61). In VQA, one can learn a prompt that, when added to the question, helps the model answer better (62). LoRA adds small learned layers to the network, so that only those are trained, drastically reducing the number of trainable parameters. However, the efficacy of parameter-efficient tuning (PET) depends on adapter capacity: if it is too small, task adaptation collapses; if it is too large, computational savings vanish. Furthermore, most studies report average metric gains but fail to provide uncertainty estimates, making it challenging to assess clinical reliability. A rigorous analysis of robustness under data drift for PET methods is currently lacking in the medical domain.
- **Instruction Tuning and RLHF:** For conversational models, final fine-tuning on instruction–response pairs is common. This can be achieved through supervised learning (utilizing high-quality question-answer pairs, possibly written by clinicians or generated and validated) or with reinforcement learning from human feedback (RLHF), where humans rank multiple outputs and a reward model is trained (58, 63). In the medical domain, RLHF is still in its early stages due to the need for expert feedback; however, some projects are exploring it, such as having radiologists rate which of two report outputs is more accurate to refine a report generator. Collecting clinician feedback is expensive, and crowd-sourced annotators risk introducing unsafe biases. Early experiments (e.g., Med-Alpaca, LLaVA-Med) demonstrate conversational fluency yet often hallucinate plausible-sounding but incorrect interpretations, underscoring the need for fact-based reward models and explainable output scoring.
- **Multitask Learning:** Instead of fine-tuning a model separately for each task, some works train a single model to handle multiple tasks simultaneously (e.g., report generation, VQA, and classification). This can be achieved by prompting, for example, by prefixing inputs with a token indicating the task. UniXGen (64) is a Unified Chest x-ray and Report Generation model that can both generate reports and even synthesize images from reports (using a VQGAN decoder). However, multitask optimization can lead to negative transfer, where improving one task actually degrades another. Curriculum schedules (progressing from simple captions to complete reports) mitigate this by shaping the optimization landscape, though empirical guidelines for task ordering are still anecdotal.
- **Curriculum and Staged Training:** A practical paradigm is to progressively train the model, starting with more manageable tasks or data and moving to harder ones. For instance, first train a model to generate impression summaries (a one-sentence summary of a report) before tackling whole report generation, so that it learns to focus on key findings. Or train on single-sentence captions from ROCO/MedICaT (65, 66), then fine-tune on multi-sentence reports. This can aid in optimization and is utilized in certain pipelines.

An example training pipeline combining many of these ideas might look like: (1) initialize visual encoder with ImageNet and text encoder with PubMedBERT; (2) perform contrastive and MLM pretraining on MIMIC-CXR and other paired data for several epochs; (3) perform supervised fine-tuning on a specific report generation dataset (like IU X-Ray or an internal dataset), possibly with a small learning rate; (4) further fine-tune using an instruction dataset where the model is given an instruction (e.g., “Write a report highlighting any pneumonia”) and the known output, to make it follow prompts; (5) evaluate on validation sets, possibly apply RLHF if needed by showing outputs to radiologists for scoring and tweaking the model. Not all studies follow all steps, but this illustrates the mix-and-match approach to maximize performance while maintaining generalizability.

It is also notable that since medical data is sensitive, federated learning and other privacy-protecting training approaches are under consideration (as hinted at in the future directions) (67). A foundation model could in principle be pretrained on decentralized hospital data by iteratively updating a global model, but this has practical difficulties. So far, most published models have been trained on publicly available datasets; however, in real-world clinical deployments, these paradigms will need to adapt to data privacy constraints. Challenges include heterogeneous hardware, non-iid data, and a lack of publicly available federated benchmarks. Without rigorous security auditing and differential-privacy guarantees, claims of “privacy-preserving” learning remain aspirational.

Table 3 aligns representative systems with their principal training objectives. Contrastive learning, masked language modeling, masked multimodal modeling, autoregressive generation, instruction tuning, and text-conditioned diffusion are treated as distinct paradigms; individual systems may combine more than one objective.

## 5 Benchmarks and evaluation

Assessing multimodal models in medicine requires attention to language quality, clinical factuality, calibration, robustness, and human factors. This section reviews common benchmarks and metrics while preserving the distinctions among report generation, VQA, retrieval, classification, and image synthesis.

### 5.1 Benchmarks and competitions

#### 5.1.1 Radiology report generation benchmarks

MIMIC-CXR and Open-I (IU X-Ray) are the standard benchmarks for automated report generation. MIMIC-CXR (41)is the larger testbed and provides a reference report for each study. Models are usually trained on one subset and tested on held-out image–report pairs, and each generated report is compared with its reference report. IU X-Ray (Open-I) dataset is much smaller (around 3,800 reports) yet has been used since the early works, which allows comparison with earlier models. Researchers often report metrics on both MIMIC-CXR and IU X-Ray to indicate generalization (68).

Gains in lexical metrics such as BLEU frequently do not translate into clinically meaningful improvement, largely because models can learn to reproduce template-like phrases and common report priors (e.g., “no acute cardiopulmonary process”) rather than image-specific findings. This copying bias means that high linguistic overlap does not necessarily indicate better diagnostic understanding or accuracy. Split leakage is another important problem: images or patients may accidentally appear in more than one of the training, validation, and test sets, which can inflate evaluation scores. The issue is especially acute for small datasets such as IU X-Ray, where the limited sample size increases the risk of overfitting and redundancy.

A further difficulty is ambiguity in the reference standard. Reference radiology reports commonly contain hedged statements, omissions, and institution-specific phrasing that reflect human variability rather than objective truth. Treating such reports as gold-standard labels carries human bias into model assessment and rewards imitation of reporting style rather than clinical accuracy. To address these challenges, researchers should use patient-level data splits, supplement language metrics with factual measures such as RadGraph F1 or CheXbert-based scores, and obtain expert judgment on stratified subsets of common and rare findings. Where possible, evaluation should use hidden, institution-held test sets to ensure fairness and prevent inadvertent data leakage.

#### 5.1.2 Medical VQA benchmarks

The main ones include VQA-RAD (69), SLAKE (70), and ImageCLEF VQA-Med series of challenges (2019–2022). VQA-RAD contains question–answer pairs for radiology images (CT, MRI, and x-ray) that focus on abnormalities. SLAKE is bilingual and includes more knowledge-based questions. The ImageCLEF VQA-Med challenges were held annually and included multiple-choice and open-ended questions. For VQA, the primary measure is accuracy (the percentage of correctly answered questions), with minor variations such as exact versus normalized matching. The VQA-Med challenges rank models by accuracy on a hidden test set. As of 2021, leading models (often ensembles or transformer-based systems) achieved accuracies of approximately 0.6–0.7 on these datasets, leaving substantial room for improvement. These benchmarks nevertheless have important limitations. Many questions are ambiguous or noisily annotated, lack adequate clinical context, or admit several valid answers. Moreover, models can exploit dataset priors, such as the imbalance between yes and no answers, rather than demonstrating visual reasoning. To address these issues, researchers should employ answer sets with synonym normalization, report calibration, and abstention metrics (e.g., coverage–risk curves), and incorporate clinician usefulness ratings on blinded subsets to assess real-world applicability.

#### 5.1.3 Captioning and retrieval benchmarks

Datasets such as ROCO (Radiology Objects in Context) and MedICaT, which comprise images and brief captions from biomedical literature, serve as benchmarks for caption generation and image–text retrieval. For captioning, metrics like BLEU and CIDEr (explained below) are reported. For retrieval (given an image, find the correct caption out of many), ranking metrics like Recall@K (e.g., Recall@10, where the correct caption falls in the top 10 retrieved) are used. The ImageCLEF 2019 caption task focused on concept detection in radiology images, which is closely related to caption retrieval. These benchmarks are, however, affected by caption noise from literature figures and domain shift between research imagery and real-world clinical scans. Mitigations include curating hard negatives, evaluating recall at K across subpopulations (e.g., device type, anatomical region, vendor), and testing cross-institutional generalization to assess robustness.

#### 5.1.4 Multimodal QA/diagnosis benchmarks

Some datasets combine images with patient data or require multi-step reasoning. PathVQA (71) and PathQA-Bench (1) are pathology VQA datasets. EndoVis 2017/2018 introduced small question sets for endoscopy images (72). While not as widely used as radiology datasets, they push models to adapt to different image types. There are also synthetic benchmarks, such as PEIR-ASK (a small Q&A dataset for anatomy). Recently, the concept of a Holistic Medical Understanding benchmark has emerged, where a model is tested on a suite of tasks (classification, captioning, VQA, etc.) to measure its generalist ability. For instance, a 2022 benchmark called MedEval proposed evaluating a model's performance across five tasks with a combined score. Yet, such composite scores can obscure regressions in safety-critical sub-tasks. To prevent this, authors should publish detailed per-task metrics, include fairness analyses across demographic and institutional slices, and report confidence intervals to ensure transparent interpretation of performance.

#### 5.1.5 Competitions and leaderboards

In 2022, a MICCAI workshop announced a public leaderboard for radiology report generation (73). This AI for Radiology Report Generation Benchmark utilized standard splits of MIMIC-CXR and introduced metrics, such as RadGraph F1 (described below), to rank submissions. Having a public leaderboard encourages consistent evaluation and has revealed that progress (as measured by BLEU or even RadGraph F1) has been steady but incremental. They also risk incentivizing leaderboard chasing, where teams focus on marginal score improvements without addressing underlying model weaknesses such as hallucinations or omissions. To ensure substantive progress, submissions should be accompanied by comprehensive model cards, detailed error breakdowns, and clinical failure analyses that highlight specific error types and their potential clinical implications. No model is yet close to radiologist-level reporting. Likewise, the VQA-Med challenge results have shown gradual improvement each year, with transformer-based models outperforming older CNN + LSTM models by significant margins since 2020.

Table 4 separates report generation from adjacent tasks. Numerical values are shown only where they are reported in the cited primary study under an identifiable protocol. RoentGen generates chest x-ray images from text, whereas LLaVA-Med is primarily a biomedical conversational and VQA system; neither is used to support a MIMIC-CXR report-generation trend. A dash or ‘not reported’ should not be interpreted as zero performance.

### 5.2 Evaluation metrics for report generation

For report generation (or image captioning in general), a variety of automatic metrics borrowed from NLP are used to compare a generated report against the reference (ground-truth) report. Each has strengths and weaknesses:

- **BLEU (Bilingual Evaluation Understudy):** Originally developed for machine translation, BLEU computes n-gram overlap between the generated text and the reference text (74). Typically, BLEU-1 through BLEU-4 are reported, measuring 1-gram up to 4-gram matches. A brevity penalty is applied to discourage overly short outputs. In report generation, BLEU has been the most common metric historically. However, BLEU mainly checks literal overlap and may not correlate with clinical correctness (a model could reorder phrases and obtain a low BLEU score despite containing correct information). An improvement in BLEU may nevertheless indicate phrasing closer to that used by radiologists.
- **ROUGE (Recall-Oriented Understudy for Gisting Evaluation):** Often ROUGE-L (Longest Common Subsequence) and ROUGE-n (n-gram recall) are used (75). ROUGE-L is helpful for measuring if the generated report covers similar content as the reference in any order, by finding the longest common subsequence of words (allowing gaps). A higher ROUGE-L score indicates that the model's output captures more key points from the reference report. In medical captions, ROUGE can be more forgiving than BLEU regarding word ordering.
- **METEOR (Metric for Evaluation of Translation with Explicit Ordering):** This metric aligns the generated and reference text by synonyms and stemmed words, then computes a score based on unigram precision, recall, and a fragmentation penalty for word order. METEOR tends to correlate better with human judgments in general domains than BLEU. In radiology, METEOR is sometimes reported; it may credit a model for using a synonym (such as “cardiomegaly” vs. “enlarged heart”), where BLEU would not (76). A METEOR score ranges 0–1, with higher being better; typical values for reports might be 0.3–0.4 for decent models.
- **CIDEr (Consensus-based Image Description Evaluation):** This computes TF-IDF-weighted n-gram similarity, initially developed for the MS-COCO captions dataset. Some medical papers utilize CIDEr to highlight important and uncommon terms. A model that mentions a rare finding present in the reference gets a boost. CIDEr has been reported in a few radiology generation works and can sometimes highlight clinically important content overlap better than raw BLEU.

BLEU, ROUGE, METEOR, and CIDEr primarily capture surface-level similarity and limited content overlap between the generated and reference reports. However, these metrics exhibit a weak correlation with clinical correctness and can be easily inflated through repetitive use of standard templates or phrases. Therefore, they should be reported only alongside clinical factual metrics, with appropriate confidence intervals (e.g., bootstrap estimates) and paired statistical tests against baselines to ensure fair comparison.

- **BERTScore:** A newer metric that uses pre-trained language model embeddings (e.g., BERT) to measure similarity between generated and reference text (77). It matches each word in the candidate to a word in the reference based on the cosine similarity of embeddings, computing the precision/recall of these matches. For medical reports, BERTScore (especially when using a clinical BERT) can capture similarity in meaning even when the wording differs. For example, “no pneumothorax seen” vs. “no evidence of collapsed lung” could score high on BERTScore due to semantic similarity, even if BLEU is low. Some studies have begun using BERTScore or related embedding metrics to more accurately assess report quality (73). However, this metric remains agnostic to factual accuracy and may reward plausible yet incorrect statements. Hence, BERTScore should be treated as a complementary metric, not a primary indicator of clinical reliability.
- **Perplexity:** If a model has a language model component, perplexity measures how well the model predicts the reference text (a lower perplexity means the model finds the text sequence more probable) (78). This is more used to evaluate how fluent the model is (lower perplexity = more confident generation). However, a model could be very fluent (have a low perplexity) but factually incorrect, so perplexity alone isn't sufficient for our purposes.
- RadGraph F1: RadGraph extracts clinical entities and relations from radiology reports and compares graph content between generated and reference text (49). It is therefore a clinical information-extraction metric, not a generic semantic-similarity score. Values depend on the report corpus, split, extractor version, preprocessing, and scoring implementation; this review does not assign a protocol-independent state-of-the-art range. RadGraph F1 can complement case-level review, but extractor errors and incomplete ontology coverage require manual adjudication of clinically important disagreements.
- **CheXpert Accuracy/CheXbert Score:** This evaluates a generated report by checking if it correctly mentions the presence or absence of specific pathologies. CheXpert is a labeler that parses text to assign labels for 14 common findings (like Edema, Atelectasis, Cardiomegaly) (57). By running the CheXpert labeler on both the generated report and the reference report, one can compute accuracy for each finding (did the model correctly mention it as present/absent/uncertain) and an overall score. CheXbert is an improved labeler (a BERT-based version) that can be used similarly (79). For evaluation, typically an F1 or exact match on the set of labels is reported. For instance, “CheXpert correctness” might refer to the percentage of the 14 conditions for which the model's report matches the ground truth report in terms of stating presence/absence. This metric directly checks clinical content but is limited to those 14 conditions (it won't penalize an error about something not in that list). While clinically meaningful, these metrics are restricted to a limited set of conditions and rely on noisy automated labelers. To ensure transparency, researchers should report performance per pathology, macro-averaged scores, and include PR-AUC when dealing with class imbalance.
- **RadCliQ:** A composite metric introduced by Rajpurkar et al. that combines several metrics (including RadGraph F1, CheXbert, BLEU, and BERTScore) into one score, attempting to reflect overall report quality (49). RadCliQ is designed to correlate with radiologist preferences. Because RadCliQ is formulated as an error score, lower values indicate better reports. Although composite indices summarize performance conveniently, their weighting can obscure trade-offs, such as improved fluency at the expense of factual accuracy. Component scores and sensitivity analyses should therefore be reported alongside the composite score.
- **Human Evaluation:** Expert review is essential for assessing clinical correctness, completeness, error severity, and workflow consequences, but it is not automatically a gold standard. A credible study should report reader specialty and experience, representative case sampling, blinding, randomization, rating criteria, adjudication, and inter-rater reliability. Blinded paired comparisons and time-on-task measures are particularly useful for assistant systems (49, 50). Human evaluation should complement, rather than replace, transparent automated metrics and case-level error analysis.

In practice, papers report a suite of metrics (BLEU, ROUGE, METEOR, possibly CIDEr, along with CheXpert or RadGraph F1) to provide a comprehensive picture. An ideal model would score highly on both language overlap (meaning it writes similarly to experts) and clinical accuracy measures. Sometimes there is a trade-off: a model could get a high BLEU by copying common phrases but might hallucinate a few findings (hurting RadGraph F1). Thus, multiple metrics are needed.

### 5.3 Evaluation metrics for VQA

For visual question answering, the evaluation is more straightforward in terms of metrics:

- **Accuracy:** If the question has a single correct answer (which could be yes/no, a specific term, or a choice), accuracy or exact match is used. For open-ended questions, typically an answer is considered correct if it matches any of a set of acceptable answers (since there can be synonyms). Datasets often include multiple reference answers per question. For example, if the question is “What abnormality is seen?” and reference answers include “pneumothorax” and “collapsed lung”, the model is correct if it outputs either. However, answer sets must include synonym mapping and case or punctuation normalization, with partial credit (token-level F1) assigned for multi-part answers. Beyond accuracy, models should be evaluated for calibration quality (using the Expected Calibration Error), selective prediction performance (coverage–risk trade-offs), and abstention utility (the benefit of deferring uncertain responses).
- **BLEU/METEOR for VQA:** Sometimes, language metrics are applied to VQA answers, especially if they are longer free-text answers. But most VQA in medicine expects short answers, so these are less common. However, if a model produces a sentence and the reference is a phrase, something like METEOR could be used to gauge similarity.
- **Specialized Metrics:** For specific VQA datasets that allow multiple-choice responses, one could use AUROC if treating them as classification problems. However, generally, VQA evaluation focuses on accuracy or the proportion of exact matches. Some tasks allow partial credit (e.g., if a question has a multi-part answer), using metrics like F1 for overlapping answer terms.

Table 5 separates lexical overlap, contextual semantic similarity, clinical information extraction, label agreement, retrieval, calibration, and expert assessment. Human evaluation is clinically important but is credible only when reader expertise, blinding, sampling, rating criteria, adjudication, and inter-rater reliability are reported.

**Table 5:** Evaluation taxonomy for multimodal medical AI. Lexical and semantic similarity, clinical information extraction, generated-text label agreement, retrieval, calibration/selective prediction, and expert assessment measure different constructs and should not be collapsed into one score.

Metric	Type	Purpose	Strengths	Limitations	Clinical Relevance
BLEU/ROUGE/METEOR	Lexical overlap	Reference-text similarity	Standardized and inexpensive	Weak proxy for factual correctness	Low alone
BERTScore	Contextual semantic similarity	Embedding-based text similarity	Recognizes paraphrase	Can reward plausible but incorrect text	Complementary
RadGraph F1	Clinical information extraction	Entity and relation agreement	Targets selected clinical content	Extractor and domain dependent	Moderate to high with adjudication
CheXpert/CheXbert label agreement	Generated-report label agreement	Agreement on selected chest findings	Clinically interpretable labels	Limited label set; automated-labeler error	Moderate to high
Human expert evaluation	Blinded clinical assessment	Correctness, completeness, severity, workflow impact	Direct clinical interpretation	Requires rigorous reader design and reliability reporting	High when well designed
Recall@K	Cross-modal retrieval	Rank a paired image or report within a candidate pool	Clear ranking endpoint	Depends on direction, candidate pool, and split	Benchmark utility only
Calibration/selective prediction	Uncertainty and abstention	Assess probability reliability and risk-coverage trade-offs	Supports safe deferral	Requires prespecified thresholds and representative prevalence	High for deployment evaluation

### 5.4 Evaluation of retrieval and other tasks

For completeness, if a model is used for image–text retrieval, metrics include Recall@K (which determines whether the correct item appears in the top-K results) and the Median Rank of the correct answer. If used for classification (like zero-shot label prediction), then classification metrics (accuracy, F1 per class) are relevant. Efficiency and speed are rarely reported in research papers but matter in practice; a model that generates a report in 2 s vs. 2 min can impact clinical workflow. Robustness tests, such as evaluation on out-of-distribution cases or adversarially perturbed images, are performed only occasionally, although they are essential for real-world deployment. Models should be evaluated under distribution shift (e.g., new hospitals or new devices), image corruptions (noise and compression), counterfactual inputs (e.g., report rewording), and time drift (year-to-year changes). Quantitative results should be accompanied by stress tests and curated failure examples that show where models are brittle.

Finally, models can be evaluated as assistants rather than autonomous systems, for example by measuring whether a radiologist's accuracy or speed improves when using model output. In simulated workflows in which clinicians revise AI-generated reports, some models have yielded reports comparable to human-only reports while requiring less time (50). Although such assessments are not yet widespread, they are essential for demonstrating practical benefit.

### 5.5 Structured quantitative synthesis and trends

#### 5.5.1 Report generation: source-traceable but non-commensurate evidence

The source audit in Table 4 shows that direct longitudinal ranking is not defensible when models address different tasks or use different evaluation protocols. R2Gen reports a MIMIC-CXR BLEU-4 value of 0.103 under its stated experimental setup (4). By contrast, RoentGen generates images from text (8), and the primary LLaVA-Med study evaluates biomedical conversation and VQA rather than MIMIC-CXR report-generation BLEU or RadGraph (13). No report-generation values are therefore attributed to these two systems. BioViL-T reports BLEU-4, ROUGE-L, CheXbert-based, and temporal-entity scores under its own MIMIC-CXR report-generation protocol (11); because its test split differs from that of R2Gen, the two sets of values are not compared directly.

Across genuine report-generation studies, lexical overlap and clinical factuality remain complementary rather than interchangeable. BLEU or ROUGE can reflect phrasing similarity, whereas RadGraph- and label-based measures assess narrower aspects of clinical content. Comparisons should therefore be restricted to studies using the same dataset, patient-level split, preprocessing, and evaluation implementation, and should include uncertainty and expert review where possible.

#### 5.5.2 Visual question answering: protocol-dependent benchmark progress

Table 6 presents source-linked VQA results and marks values that are not reported in the cited primary studies. VQA-RAD, SLAKE, PathVQA, and ImageCLEF VQA-Med differ in answer format, split construction, normalization, and clinical context. Consequently, apparent differences across models or datasets should be interpreted as benchmark-specific rather than as a common maturity scale. LLaVA-Med is discussed here because its primary contribution includes biomedical VQA (13), not because it is a radiology report-generation system.

**Table 6:** Source-linked medical visual question answering (VQA) evidence. Acc, accuracy; NR, not reported in this audit; N/A, not applicable. Results should be compared only under the same dataset split, answer format, normalization, and evaluation protocol.

Model	Arch.	VQA-RAD (%)	SLAKE (%)	PathVQA (%)	VQA-Med 2019 (%)	Training	Source/protocol
MMBERT (2021) (9)	Unified encoder	62.5	NR	NR	NR	Multimodal pretraining	Study-specific split; compare only under the same protocol
MedViLL (2022) (10)	Unified encoder	67.8	NR	NR	NR	Vision–language pretraining	Primary paper reports multiple downstream tasks
LLaVA-Med (2023) (13)	Multimodal LLM adapter	NR in this audit	NR in this audit	NR	NR	Biomedical instruction tuning	Primary study reports separate closed/open VQA protocols; no pooled accuracy is attributed
Med-Flamingo (2023) (3)	Few-shot multimodal LLM	NR	NR	NR	NR	Few-shot prompting	Primary evaluation includes generative VQA and clinician ratings; no unsupported table values added
Visual Med-Alpaca (2023) (53)	Multimodal LLM	NR	NR	NR	NR	Parameter-efficient tuning	No standardized values attributed here
PathChat (2024) (15)	Pathology multimodal assistant	N/A	N/A	NR	N/A	Pathology instruction tuning	Primary study is not treated as a PathVQA leaderboard entry
XrayGPT (2023) (14)	CXR conversational VLM	NR	NR	N/A	N/A	Instruction tuning	No standardized VQA-RAD value attributed without a matching source
ImageCLEF VQA-Med baselines (80–82)	Various	N/A	N/A	N/A	See annual reports	Challenge protocols	Annual datasets and scoring differ; no pooled baseline range

Values are retained only where they are attributable to the cited study. Dataset splits, open/closed question composition, answer normalization, and scoring implementations differ across papers.

#### 5.5.3 Retrieval: strong benchmark results with protocol caveats

Image–text retrieval systems often report high Recall@K, but the value depends on the dataset, candidate-pool size, retrieval direction, and test split. Table 7 therefore retains the values reported in the cited papers while adding source identifiers and an explicit non-comparability note. BiomedCLIP was pretrained on PMC-15M, a 15-million-pair corpus, which is distinct from the approximately 1.6-million-pair PMC-OA resource used in PMC-CLIP (12, 83).

**Table 7:** Source-linked image-text retrieval results. Recall@K is protocol dependent. Dataset, split, retrieval direction, candidate-pool size, and preprocessing must match before systems are compared.

Model (Year)	Architecture	Dataset	Recall@1	Recall@5	Recall@10	Direction(s) Reported	Training Scale
ConVIRT (2022) (6)	Dual-Encoder	MIMIC-CXR	—	—	0.81	Both	377k images
GLoRIA (2021) (5)	Dual-Encoder (Global-Local)	MIMIC-CXR	0.51	0.78	0.87	Both	377k images
MedCLIP (2022) (7)	Dual-Encoder	MIMIC-CXR + Custom	0.47	0.76	0.85	Both	217k pairs
BiomedCLIP (2023) (12)	Dual-Encoder	PMC-15M	0.58	0.82	0.89	Both	15 million image–text pairs
RET-CLIP (2024) (43)	Dual-Encoder (Ophthalmology)	Retinal images	—	—	0.83	Both	Domain-specific
Mammo-CLIP (2024) (44)	Dual-Encoder (Mammography)	Mammography exams	—	—	0.81	Both	Domain-specific

Protocol caveat: Retrieval values are not directly rankable across different datasets, candidate-pool sizes, directions, splits, and preprocessing pipelines. Each row should be interpreted with its cited primary study.

These results support the usefulness of contrastive pretraining for retrieval, but they do not demonstrate human-equivalent performance or clinical deployment readiness. Head-to-head conclusions require a shared dataset, candidate pool, retrieval direction, and evaluation code.

#### 5.5.4 Cross-task evidence summary

Table 8 is an exploratory evidence summary, not an AI/human ratio or clinical-readiness ranking. Recall@K, VQA accuracy, classification F1, and RadGraph F1 measure different constructs and cannot be normalized into a single clinically meaningful scale.

**Table 8:** Exploratory cross-task evidence summary (not a clinical-readiness scale).

Task Category	Representative Systems	Common Metric	Human Comparator	Principal Comparability Limitation	Evidence Maturity
Image–text retrieval	BiomedCLIP (12)	Recall@K	Not standardized	Dataset, candidate pool, direction, and split	Benchmark evidence
Closed-set VQA	LLaVA-Med (13)	Accuracy/exact match	Not standardized	Answer set, normalization, and split	Benchmark evidence
Open-ended VQA	LLaVA-Med (13)	Exact match, token F1, or expert score	Study-specific	Multiple valid answers and reader protocol	Early clinical evidence
Classification	Contrastive medical VLMs	AUROC/F1	Task-specific	Label definition, prevalence, threshold, and external validity	Task-specific evidence
Report generation	R2Gen (4) and later task-specific systems	BLEU, RadGraph, label agreement, expert review	Study-specific	Protocol heterogeneity and safety-critical error severity	Early clinical evidence

Interpretation: Metrics across rows measure different constructs. No ratio or label in this table should be interpreted as evidence of clinical deployment readiness.

Instead, the synthesis distinguishes the type of evidence available for each task, the principal comparability limitation, and the extent of clinical validation. Across tasks, retrospective benchmark performance should be considered only one component of evidence alongside calibration, external validation, subgroup safety, workflow impact, and prospective human-factor evaluation.

#### 5.5.5 Methodological heterogeneity and reproducibility

Within the 13-study evidence matrix, 4 studies reported BLEU/ROUGE (31%), none reported RadGraph F1 (0%), 6 used CheXpert- or CheXbert-derived label evaluation (46%), 1 reported BERTScore (8%), 4 included human expert evaluation (31%), and none reported inference latency or throughput (0%). The denominator is 13 for every proportion. Task-inapplicable entries remain in the denominator because the table audits reporting across the displayed heterogeneous sample; they are marked N/A rather than treated as zero performance.

These proportions are descriptive and do not establish temporal trends or field-wide prevalence. Training compute, hardware, energy use, and inference latency are separate variables and should be reported separately. For reproducibility, future comparative tables should specify the dataset version, patient-level split, preprocessing, image resolution, report section, evaluation code, uncertainty interval, and primary source for every value.

In summary, medical vision–language models require multidimensional evaluation. Lexical metrics describe overlap with reference text; contextual metrics describe semantic similarity; graph- or label-based metrics assess selected aspects of clinical content; and expert studies evaluate acceptability and workflow consequences. No single metric or cross-task ratio establishes clinical readiness.

## 6 Public datasets

A variety of publicly available datasets have catalyzed research on multimodal medical AI. These datasets provide images paired with text (reports, captions, or QA) and serve as both training foundations for models and evaluation benchmarks. We summarize the most prominent datasets from 2020 to 2025 relevant to vision–language modeling, organized by application domain (radiology, general biomedical, VQA, etc.). Table 9 below provides an overview of key datasets, including their modality, size, and use cases.

**Table 9:** Key public datasets for vision–language medical AI.

Dataset (Year)	Modalities	Size (pairs)	Description/Use
**MIMIC-CXR** (41)	X-ray + Report (EN)	377,110 images, 227,835 reports	Largest chest x-ray dataset with free-text radiology reports. Widely used for report generation and pre-training.
**MIMIC-CXR-JPG** (84)	X-ray + Report (EN)	377k images (JPEG)	Preprocessed version of MIMIC-CXR with JPEG images for easier use; same reports as MIMIC-CXR.
**IU X-Ray (Open-I)** (68)	X-ray + Report (EN)	7,470 images, 3,955 reports	Indiana Univ. chest x-rays with reports. Standard benchmark for report generation. Smaller scale.
**CheXpert** (57)	X-ray + Labels (EN)	224,316 images, 14 labeled observations	Stanford chest x-ray dataset with 14 pathology labels (including uncertainty labels). Used for classification and as a source for the CheXpert labeler. (No complete reports publicly available).
**PadChest** (40)	X-ray + Report (ES/EN)	160,000 images, ∼110,000 reports	Spanish hospital CXR dataset with reports (Spanish) and labels. Useful for multilingual learning and fairness.
**ROCO** (66)	Misc. Medical Images + Caption (EN)	∼81,000 images, 81,000 captions	Radiology and other medical images from PubMed articles with associated figure captions. Used for pre-training (e.g., PubMedCLIP).
**MedICaT** (65)	Med. images + Caption + (refs)	217,060 figures, captions from ∼131k papers	Extended ROCO with additional textual context (subfigure references, etc.). Useful for pre-training and captioning tasks.
**PMC-OA/PMC-CLIP** (2023) (83)	Med. images + Caption (EN)	Approximately 1.6 million image–caption pairs	Biomedical figures and captions from PubMed Central Open Access; used for PMC-CLIP. Distinct from PMC-15M.
**VQA-RAD** (69)	Radiology images + Q&A (EN)	3,515 questions; 315 images	Earliest radiology VQA dataset. Qs about CT, MRI, x-ray with short answers. Small but popular for evaluating VQA models.
**SLAKE** (70)	Mixed images + Q&A (EN/ZH)	642 images, 14k Q&A pairs	Bilingual (English/Chinese) VQA dataset covering various modalities. Questions require reasoning and some clinical knowledge.
**PathVQA** (71)	Pathology images + Q&A (EN)	4,998 images, ∼32,799 Q&A	Pathology VQA dataset based on educational/pathology images; it should not be described as a whole-slide-image benchmark.
**VQA-Med 2019 (**80)	MedPix images + Q&A (EN)	4,200 images, 15,292 Q&A	Challenge dataset (ImageCLEF, 2019) with questions about radiology images from the MedPix database. Primarily used for competition, multiple-choice, and open questions.
**VQA-Med** (Abacha, 2020)	MedPix images + Q&A (EN)	5,000 images, 5,000 Q&A	ImageCLEF 2020 VQA challenge data, fewer questions, more focused on specific modalities.
**VQA-Med (**82)	MedPix images + Q&A (EN)	5,500 images, 5,500 Q&A	ImageCLEF 2021 challenge, with a similar format and incremental additions.
**MIMIC-NLE** (85)	X-ray + Natural Language explanations (EN)	38003 images image-NLE pairs or 44,935 image-diagnosis-NLE triplets.	A subset of MIMIC for Natural Language Explanation: models must justify a diagnostic classification with a text explanation. Useful for training models to provide reasoning (used in some VQA works).
**CXR-PRO** (86)	X-ray + modified Report	374k reports (from MIMIC-CXR)	A processed version of MIMIC reports with references to prior studies removed. Created to eliminate misleading context for models (avoiding learning to hallucinate “Since previous exam…” statements).
**MS-CXR** (42)	X-ray + annotated findings	∼1.2k image–sentence pairs with bounding boxes (MIMIC-CXR subset)	Radiologist-verified bounding boxes paired with report phrases for eight cardiopulmonary findings, drawn from MIMIC-CXR and released with BioViL (42). Used to evaluate phrase grounding and localization.
**PMC-15M/BiomedCLIP** (2023) (12)	Biomedical figures + captions (EN)	15 million image–text pairs	Large-scale scientific image–text corpus used to pretrain BiomedCLIP; distinct from PMC-OA/PMC-CLIP.

### 6.1 Usage

Many of these datasets are used in conjunction with one another. For example, a model might pre-train on PMC-OA (general biomedical captions) and MIMIC-CXR, then fine-tune on IU X-Ray for evaluation. The diversity of data sources is important: radiology reports tend to have a particular style (formal, complete sentences), whereas figure captions from journals are often telegraphic and assume a caption-reader context (e.g., “Figure 3: T2 MRI showing lesion…”). Another style is VQA datasets, which deal with brief responses. All these styles can be fed into a model to make it more flexible. Other models synthesize combinations, such as the use of a report to generate Q&A pairs to enhance VQA training.

### 6.2 Multilingual data

PadChest (Spanish) and a few bilingual datasets (e.g., SLAKE, with Chinese) are important because they require models to handle non-English text. English still dominates the field, but these datasets allow preliminary multilingual experiments. Med-UniC (87), for example, performs cross-lingual vision–language pretraining on English (MIMIC-CXR) and Spanish (PadChest) radiology reports to reduce language-related bias.

### 6.3 Task-specific splits

Several datasets provide official train, validation, and test partitions, particularly the ImageCLEF VQA-Med challenges. Researchers should use these partitions and report any deviations. PhysioNet also provides recommended patient-level splits for MIMIC-CXR that support more consistent evaluation.

### 6.4 Data quality and limitations

Despite their importance, these datasets have limitations. Radiology reports may be noisy, with typographical errors, inconsistent structure, and references to prior examinations that the model cannot observe. Resources such as CXR-PRO remove some of this content (e.g., comparisons with previous images) to reduce unobservable context. Image quality also varies: MIMIC-CXR DICOM images are high-resolution, but many studies use the compressed JPG version, often further downsampled to reduce training time, which may lose subtle details. VQA datasets often contain questions generated by experts, which can be quite challenging, sometimes requiring external knowledge (e.g., “What is the most likely diagnosis given the image and patient age?” necessitates knowledge of diseases common at specific ages). This means that a pure image-to-text model might struggle if it lacks that knowledge.

### 6.5 Emerging datasets

Newer resources combine modalities further, for example by linking chest radiographs with structured labels, report sections, and electronic health record data (e.g., research datasets that link ICU records with radiographs). These are not yet mainstream, but they point to future combined-modality training data (see Future Directions).

For our purposes, the datasets listed in Table 9 have been the backbone of vision–language foundation model training and evaluation. MIMIC-CXR has played a role comparable to that of ImageNet for medical multimodal learning, enabling many groups to explore ideas without data-collection barriers. Similarly, the VQA-Med challenges provided a common yardstick for question answering. Table 10 summarizes key datasets in medical vision–language research, including their modalities, strengths, limitations, and ethical considerations.

**Table 10:** Critical appraisal of key public datasets for vision–language medical AI.

Dataset	Primary Modality/Language	Strengths	Limitations	Bias/Ethical Notes
**MIMIC-CXR (2019)**	Chest x-ray + English reports	Largest paired dataset; standardized benchmark; rich metadata	Single-center (U.S. ICU); adult-only; high report redundancy	Geographical bias (North America); de-identified PHI but limited consent scope
**IU X-Ray (Open-I)**	Chest x-ray + English reports	Easy benchmarking; legacy comparability	Very small (<4 k reports); prone to data leakage	Lacks demographic diversity; limited licensing metadata
**CheXpert (2019)**	X-ray + 14 labels	Label consistency; uncertainty modeling	No full reports; label noise from rule-based parser	Potential gender and age imbalance
**PadChest (2020)**	X-ray + Spanish/English reports	Multilingual; high-quality annotations	Single institution; partial translations	Helps mitigate language bias, but still Spain-centric
**ROCO/MedICaT**	Biomedical figures + captions (EN)	Diverse modalities; open license	Caption noise; weak clinical context	Potential copyright ambiguity for some images
**PMC-OA (2023)**	1.6 M figures + captions (EN)	Massive scale for foundation pre-training	Heterogeneous format; weak supervision	License heterogeneity (CC mix); bias toward academic English
**VQA-RAD/SLAKE/VQA-Med**	Radiology/mixed modalities + Q&A	Evaluates reasoning; standard leaderboards	Small size; ambiguous questions	Western lexical bias; limited clinician validation
PathVQA (71)	Pathology images + Q&A (EN)	Expands evaluation beyond radiology	Small, task-specific images; not a whole-slide-image benchmark	Domain-specific language bias
**MIMIC-NLE (2022)**	X-ray + natural-language explanations	Introduces an explainability benchmark	Derived from MIMIC → same demographic bias	Needs ethical review for explanatory text use

Although these resources have broadened access to research, together they reinforce a narrow demographic and linguistic scope dominated by North American, English-language, adult chest radiography. Vision–language models trained on them may therefore generalize poorly to pediatric, non-Western, or multilingual populations. PadChest and SLAKE add Spanish and Chinese, respectively, but their regional scope remains limited, which restricts cross-cultural robustness. A second recurring issue is annotation reliability. Automated labelers (e.g., the CheXpert labeler) and weakly supervised caption extraction introduce systematic noise that can propagate through training, producing spurious correlations and hallucinated findings. Manual expert annotation is more precise but costly, creating a trade-off between scale and precision.

Licensing and privacy arrangements also differ considerably. MIMIC-CXR is de-identified and available through a credentialed data-use process, whereas literature-derived datasets (e.g., ROCO, MedICaT, and PMC-OA) aggregate biomedical figures under mixed or ambiguous licenses. These differences complicate commercial and cross-institutional reuse and raise questions about the ethical limits of open-access AI training in medicine. Bias and representativeness should also be examined directly. The predominance of data from high-income countries (mainly the United States and Spain) limits exposure to diverse disease distributions, imaging equipment, and reporting styles. In addition, English-language clinical narratives carry lexical and cultural assumptions that may not transfer to other healthcare systems. Models that are not deliberately balanced are prone to systematically lower performance in underrepresented groups, which is a significant obstacle to equitable deployment.

Progress toward globally trustworthy vision–language models will require federated, multi-institutional data resources that protect patient privacy while increasing demographic and geographic diversity. This, in turn, requires cross-institutional validation supported by harmonized metadata standards, including consistent use of DICOM headers and demographic descriptors. Multilingual report alignment, supported by high-quality translation and shared ontologies such as RadLex and SNOMED CT, may help bridge linguistic and terminological differences between healthcare systems. Dataset documentation should also include bias audits and fairness benchmarks to reduce systematic inequities in data representation and model performance. Finally, strong ethical governance is needed so that the use of medical text and images is transparent and consistent with privacy law and license terms.

Overall, public datasets have enabled substantial progress in multimodal medical AI, but demographic imbalance, annotation noise, and ethical ambiguity remain unresolved. Future dataset efforts should prioritize representative sampling, validated high-quality annotation, standardized licensing, and federated data-sharing protocols so that foundation models are clinically trustworthy and globally inclusive, not only high-performing.

## 7 Challenges

Despite considerable progress, substantial work remains before vision–language foundation models can be used reliably in clinical practice. The difficulties arise from both technical barriers and the complexity of healthcare systems. This section summarizes the key challenges identified in recent studies and the mitigation strategies proposed in the literature:

### 7.1 Data availability and quality

Foundation models require large, diverse training datasets, whereas medical data are frequently sparse, siloed, or unlabeled. Annotated multimodal datasets, particularly outside radiography, remain scarce (2). Additionally, any existing dataset may have mistakes or gaps (e.g., the text of the report may not be a true description of all findings in the image). This scarcity may result in models that generalize poorly. Possible remedies include self-supervised learning to exploit unlabeled data (e.g., images without reports) and cross-institutional collaboration to build federated datasets. Synthetic data (Section 8.5) and transfer learning from general-domain data may also help. Additionally, mandatory dataset datasheets and bias audits should be implemented to document provenance, missingness, and demographic balance. Cross-institutional validation and multi-site testing, ideally across low, middle, and high-income settings, should become a requirement for clinical VLM benchmarking. Fundamentally, however, increasing the size and diversity of high-quality medical multimodal corpora (while respecting privacy) is an ongoing challenge for the community.

### 7.2 Bias and fairness

Medical datasets may contain demographic, disease-prevalence, institutional, and language biases (87). Evaluation should therefore report subgroup performance and uncertainty rather than assuming that aggregate accuracy transfers across populations. PadChest can support Spanish-language analysis (40), while FairCLIP proposes fairness-aware representation learning (88). CheXpert++ is a faster differentiable approximation of the CheXpert labeler (89), not a fairness-oriented dataset. Fairness claims require explicit demographic labels, predefined subgroup analyses, and external validation.

### 7.3 Hallucinations and factual consistency

A major concern is that generative models can produce clinically plausible statements that are not supported by the image. Such errors include invented findings, omitted abnormalities, incorrect negation, and mis-localization. Mitigation strategies include evidence grounding, uncertainty-aware abstention, post-generation consistency checks, and blinded expert adjudication. RadGraph and label-based measures can assist evaluation, but they should not replace case-level clinical review.

### 7.4 Interpretability and trust

Attention maps, saliency maps, localization overlays, and generated reasoning traces are candidate user interfaces, not proof that a model has revealed the causal basis of its output (16, 17). A fluent rationale may be *post hoc* or hallucinated, and attention weights may change without a corresponding change in the prediction. Evaluation should therefore test localization against expert annotations, use perturbation and counterfactual tests, assess concept-level evidence, report uncertainty and abstention behavior, and determine in blinded clinician studies whether the interface improves error detection without increasing automation bias. Explanation claims should specify the target user, intended decision, validation endpoint, and known failure modes.

### 7.5 Generalization and robustness

Medical images from different sources (hospitals, devices) vary. A model trained on one hospital's x-rays may struggle with those from another due to differences in imaging protocols or patient demographics. Additionally, unusual cases (rare diseases or uncommon presentations) are challenging to learn if they rarely appear in training. This raises the risk that a model might perform well on test data similar to the training data (e.g., common chest findings) but fail on edge cases or new modalities. Moreover, some models might be overfit to the style of reports in training data, e.g., many radiology reports follow templates, so a model might learn to produce template-like text without truly understanding the image (and it could break when encountering a scenario where the template doesn't fit). Potential mitigations include prioritizing diverse training data and improving robustness through augmentation (e.g., adding mild noise) or simulation of other acquisition conditions. Ensembles or uncertainty estimation can flag when a model is uncertain, which often coincides with out-of-distribution inputs. Continual learning, in which models are updated with new data (e.g., during a new disease outbreak such as COVID-19, which was absent from earlier training data), is also of interest, but updates should be incremental to avoid loss of prior knowledge.

### 7.6 Evaluation limitations

As mentioned in the evaluation section, metrics do not provide the complete picture. One problem is how to design evaluation protocols that reflect clinical utility. For example, a model may achieve a good RadGraph F1 score yet miss an important finding such as a pneumothorax, which would be unacceptable. Existing metrics may not reflect the severity of such errors. One mitigation is error-severity grading, in which omission of a life-threatening finding is penalized more heavily than a minor discrepancy. Evaluation could also be hierarchical: first checking whether critical conditions are correctly identified (e.g., no missed pneumothorax or false cancer diagnosis) and then reporting overall metrics. Human-in-the-loop assessment, as in the study in which clinicians edited AI-drafted reports (50), provides richer feedback on model performance, but such assessments are resource-intensive and difficult to scale during routine model development.

### 7.7 Regulatory, ethical, and governance considerations

The 2021 GMLP principles were issued jointly by the U.S. FDA, Health Canada, and the U.K. MHRA and were subsequently harmonized through the final IMDRF GMLP document in 2025 (90, 91). GMLP addresses total-product-lifecycle practice for machine-learning-enabled medical devices; TRIPOD+AI and CLAIM are reporting guidelines for prediction-model and medical-imaging AI studies, respectively (92, 93); and the EU AI Act is a binding, risk-based legal framework whose applicability depends on jurisdiction, intended purpose, and product classification (94). These sources are complementary, not interchangeable, and none alone establishes authorization for clinical use.

A governance program should document data provenance, intended use, version history, update procedures, human oversight, performance monitoring, calibration drift, subgroup safety, and incident response. The specific regulatory pathway depends on jurisdiction, intended medical purpose, and whether the model functions as medical-device software.

Developers and deploying institutions should also maintain privacy, cybersecurity, and access controls consistent with applicable law. Model cards and risk summaries should state the model's scope, data sources, known limitations, prohibited uses, update policy, and evidence supporting any clinical claim.

### 7.8 Integration with clinical workflow

Clinical value depends on how a system changes decisions, workload, and error recovery, not on output fluency alone. Studies should define who reviews and can override model output, how uncertainty and abstention are displayed, how critical findings are escalated, and how automation bias is monitored. Prospective or simulated-workflow studies should compare clinician accuracy, time, workload, and failure recovery with and without assistance. Draft-report and highlighting interfaces require separate validation because they create different human-factor risks.

### 7.9 Computational sustainability

Training billion-parameter VLFMs is expensive in energy and hardware, raising concerns about environmental impact and accessibility. Such computational inequity risks concentrating innovation in a few well-resourced institutions. Possible solutions include model compression, knowledge distillation, parameter-efficient tuning (e.g., LoRA and adapters), and public repositories of shared pre-trained backbones. Carbon-accounting protocols for model training, in the spirit of the Green AI movement, can encourage environmental responsibility. Lightweight, task-specific clinical adapters are cheaper and less energy-intensive and may support sustainable deployment in hospitals.

### 7.10 Privacy and security

Medical information is confidential. General-domain foundation models are typically trained on data collected from the web, whereas in medicine patient data may be highly sensitive even in aggregated form. Federated learning (training models across institutions without sharing raw data) is a promising solution, but technically challenging for such complex models (67). Another concern is whether a model could inadvertently leak patient information from its training set through memorization. De-identification of training data, as in MIMIC-CXR, mitigates this risk, but models can still memorize specific images or text verbatim if not properly regularized. Security evaluation, including testing against adversarial attacks in which an image is subtly altered to mislead the model, is an emerging challenge; small perturbations are known to change the outputs of medical image classifiers, and similar vulnerabilities may affect report-generating VLMs.

### 7.11 Scaling and efficient deployment

This subsection focuses on deployment constraints that are distinct from the environmental and access concerns discussed in Section 7.9. Studies should report parameter count, memory requirements, hardware, batch size, latency, throughput, and performance after compression or quantization. These details determine whether a model can operate reliably within hospital infrastructure and time-critical workflows.

The challenges facing vision–language foundation models are ethical, environmental, and regulatory as well as technical. Addressing them requires collaboration among data scientists, clinicians, ethicists, and policymakers to develop transparent, auditable, and sustainable systems. Such coordinated effort is needed for medical VLFMs to progress from experimental prototypes to clinically dependable tools.

Section 8 discusses ongoing and potential approaches to overcome these challenges, such as integrating EHR data to provide more context (addressing some hallucinations), using federated learning for privacy, employing diffusion models for generating synthetic data to boost diversity, and developing generalist models that can handle many tasks with higher robustness.

## 8 Future directions

This section outlines a structured research roadmap for medical vision–language foundation models, focusing on directions that could improve capability, safety, and clinical utility while addressing current limitations such as bias, hallucination, and limited interpretability:

### 8.1 Cross-modal reasoning and knowledge grounding

Current medical VLMs primarily align vision and language through statistical co-occurrence, rather than relying on causal or clinical reasoning. The next generation must develop explicit cross-modal reasoning, integrating medical knowledge bases and ontologies to ground model predictions in medical knowledge. Promising approaches include retrieval-augmented generation (RAG) (95), where external medical literature or past imaging cases are dynamically retrieved to support factual outputs, as demonstrated for retrieval-augmented biomedical VQA (102) and chest x-ray report generation (106), and graph-based reasoning, in which clinical entities and relations (e.g., disease–anatomy–finding) are represented as knowledge graphs. Knowledge-guided approaches, such as RadGraph-informed report generation, suggest that structured medical context may reduce hallucinations and improve interpretability. Ultimately, the goal is to build systems that can justify findings (e.g., “right lower-lobe opacity; in the context of fever and elevated CRP, consistent with pneumonia”) rather than merely describe them.

### 8.2 Temporal and longitudinal modeling

Most current datasets and benchmarks treat cases as static images. Clinical reasoning, however, depends on disease progression, treatment response, and serial imaging patterns.

Future VLMs should include temporal encoders that can process image sequences (e.g., serial chest x-rays or echocardiography videos) together with evolving text such as follow-up notes or laboratory trajectories. Temporal pretraining that uses the prior chest radiograph and report, as in BioViL-T (11), is an early step in this direction. Hybrid architectures that combine transformers with recurrent or attention-based temporal modules may support dynamic monitoring of patient progress. Temporal reasoning also underpins early-warning systems in which an AI flags deterioration by comparing new scans with baseline images. Moving from snapshot interpretation to trajectory-level understanding is necessary for realistic clinical use.

### 8.3 Integration with electronic health records (EHR) and multi-omics data

A longer-term goal is to create holistic AI assistants that consider not only images and reports but also patient history, lab results, medications, genomics, proteomics, metabolomics, and other EHR data (96). For example, an AI system reading a chest x-ray could consider the patient's clinical notes, vital signs, and laboratory results to contextualize its interpretation and produce a more individualized report. Relevant building blocks include large clinical language models such as GatorTron, which can encode unstructured EHR text, and the generalist medical AI (GMAI) concept, which envisions models that combine images with structured and unstructured clinical data (97). Combining EHR and multi-omics data may reduce misdiagnoses by supplying essential context; for example, knowing that a patient is immunocompromised could make a model more alert to opportunistic infections on imaging.

### 8.4 Federated and privacy-preserving learning

As noted above, data silos and privacy concerns restrict access to training data. Federated learning (FL) could be used to train future foundation models across hospitals (98). In FL, each institution's data remain behind its firewall and only model updates are aggregated. This has been tested on simpler medical models (such as federated COVID-19 image analysis). Scaling FL to large VLMs is difficult because of communication cost and heterogeneity between sites, but work on improved aggregation (e.g., FedAvg variants) and differential privacy is in progress (99). Another approach is split learning, where the image encoder can run on-site at the hospital, and only the intermediate embeddings (not the raw image) are sent to a central server for further processing, thereby reducing privacy risk. Regulatory frameworks like GDPR and HIPAA drive interest in such methods, because training on private data without exposing it would make large amounts of currently inaccessible data usable. Additionally, synthetic data generation (discussed next) can complement privacy efforts by creating shareable data.

### 8.5 Synthetic data and diffusion models

Diffusion models and other generative models (GANs, etc.) can create realistic medical images given specific inputs. A future direction is to use these models to generate synthetic image–text pairs for augmenting training. Early works have used GANs to augment data for classifiers; diffusion models (e.g., Stable Diffusion variants adapted to radiology) produce higher-fidelity images and may be able to generate labeled datasets for underrepresented cases. Proof-of-concept studies already exist: RoentGen adapts a latent-diffusion model to generate chest radiographs from radiology text prompts (8), and cascaded latent diffusion models have been used for high-resolution chest x-ray synthesis (100). For report generation specifically, one could generate additional training examples of, say, “positive pneumothorax” cases to ensure the model learns that scenario well, without needing real patients with pneumothorax. However, synthetic data must be used carefully, as it can introduce its own biases or artifacts if it is not truly representative of the real world. Nonetheless, as diffusion models improve, they might also be integrated directly into the vision–language pipeline.

### 8.6 Generalist medical AI systems (GMAI)

The concept of Generalist Medical AI is to have one model handling a broad spectrum of tasks and modalities in healthcare. Instead of training separate models for radiology, ophthalmology, dermatology, and other specialties, a single foundation model could be trained on all of them, thereby sharing knowledge across domains. The Nature perspective by Moor et al. outlines such a GMAI that can interpret different combinations of inputs (images, labs, text) and produce diverse outputs (diagnoses, reports, referrals, patient-friendly explanations) (96). Achieving this would likely involve massive multimodal multi-task training, essentially combining all sorts of data into one model. Although this goal is ambitious, there are early signs of progress: models such as BiomedCLIP span multiple biomedical image types, and general-purpose large language models are being evaluated on medical tasks, including image-based questions and generalist biomedical models such as Med-PaLM M interpret clinical language, imaging, and genomic data with a single set of model weights (104), although their medical-imaging performance is not fully characterized. One specific direction is multimodal LLMs specialized for medicine that accept heterogeneous inputs (potentially an entire patient record, including images) and answer queries or offer suggestions. Agent-based systems, in which a language model orchestrates specialized vision tools (103), could extend this further, for example by proposing follow-up tests after reviewing a patient's chart and imaging, or by checking consistency between pathology and radiology reports; such autonomy would require particularly rigorous safety evaluation. Generalist models trained on both pathology and radiology data might also reveal cross-domain associations, such as links between imaging and histological features, that single-domain models cannot capture.

### 8.7 Improved evaluation and validation frameworks

As models become more capable, evaluation must keep pace, and benchmarking frameworks that assess models in more realistic settings are needed. For example, rather than scoring single generated reports, future benchmarks could simulate the workflow: the model drafts a report, an expert corrects it, and the time saved or the difference in diagnostic accuracy with and without the model is measured. Another concept is longitudinal evaluation, in which a model receives a patient's images over time and is assessed on its ability to track changes or detect new findings. This is clinically relevant, because radiologists routinely compare current and prior examinations, but it is seldom tested; MS-CXR-T, released with BioViL-T (11), is one of the few benchmarks that targets temporal semantics. Validation should also include more prospective studies, such as clinical trials that measure patient outcomes or clinician efficiency.

### 8.8 Regulatory and Ethical Frameworks

Translation studies should distinguish among lifecycle practice, reporting guidance, and legal obligations. FDA/Health Canada/MHRA GMLP informs AI/ML medical-device development (90); TRIPOD+AI and CLAIM support transparent reporting (92, 93); and the EU AI Act imposes risk-based legal requirements within its jurisdiction (94). Future work should document intended use, human oversight, data governance, bias controls, post-deployment monitoring, and change-management procedures.

### 8.9 Specialized modalities and new domains

So far, this review has mainly discussed 2D images and text. Future work is likely to extend multimodal models to 3D imaging (CT and MRI volumes). Processing a complete CT series, which may contain hundreds of slices, is a substantial technical challenge that requires efficient volumetric encoders or summarization methods. Early work in this direction includes vision–language foundation models for 3D medical imaging (101). Models that generate reports directly from CT volumes are a natural next step, although current attempts remain early. Ophthalmology is another area in which multimodal models combining retinal imaging and clinical information could help predict systemic conditions. Video with language is a further frontier (e.g., echocardiography or surgical video): an AI system could review an ultrasound clip and produce a structured report of measurements and observations. Early work has combined video frames and text (e.g., EndoVis surgical data), and this area is likely to expand as computing resources grow (72).

### 8.10 Sustainability and democratization of medical VLMs

Future work should reduce the computational and access burden of VLFMs through parameter-efficient tuning, distillation, quantization, shared evaluation infrastructure, and transparent compute reporting. Environmental impact, affordability, and deployment feasibility should be assessed separately rather than collapsed into a single efficiency claim.

### 8.11 Human–AI collaboration and education

Human–AI interfaces should be designed for active collaboration rather than passive automation. In an emergency-department reader study, physicians rated generative-AI chest radiograph reports as similar in quality to radiologist reports and higher than teleradiology reports (105). Future systems should support interactive, conversational workflows in which clinicians can ask the model for supporting evidence or counterexamples and provide corrective feedback. Parallel efforts in AI literacy and clinical education will help professionals understand the strengths and weaknesses of such systems and calibrate their trust. Simulation-based training environments may also be incorporated into radiology and pathology residency programs.

The research roadmap for medical vision–language models extends beyond incremental accuracy gains toward building context-aware, interpretable, sustainable, and equitable systems. Progress in multimodal reasoning, longitudinal data integration, and ethical governance will largely determine whether VLFMs move from experimental prototypes to clinically dependable and widely accessible tools.

## 9 Minimum clinical validation and reporting standard

The following checklist is proposed as a minimum for studies that make clinical-performance or deployment claims. It is a reporting and evidence framework, not a substitute for jurisdiction-specific regulatory review.

### 9.1 Data provenance and study design

Report dataset source, licensing, inclusion and exclusion criteria, patient-level train/validation/test splits, preprocessing, report sections, missing data, demographic composition, and measures taken to prevent leakage. Clearly separate internal development data from external validation data.

### 9.2 Task-specific performance and uncertainty

State the intended task and primary endpoint before analysis. Report clinically meaningful metrics alongside language metrics; include confidence intervals, calibration or selective-prediction measures where applicable, per-finding results, and an error-severity analysis covering hallucinations, omissions, negation, and localization errors.

### 9.3 External, subgroup, and robustness validation

Evaluate at an independent institution or clearly justify why this is not possible. Report performance across relevant demographic, device, site, and disease-prevalence subgroups, and include distribution-shift or stress tests appropriate to the intended use.

### 9.4 Human evaluation and workflow impact

Describe reader expertise, sample selection, blinding, randomization, rating criteria, adjudication, and inter-rater reliability. For assistant systems, measure whether the model improves clinician accuracy, time, or workload and document automation bias and failure-recovery procedures.

### 9.5 Compute, transparency, and lifecycle monitoring

Report model size, training and inference hardware, memory use, latency, throughput, software version, and relevant energy or cost measures. Provide an intended-use statement, model card, known limitations, change-management plan, post-deployment monitoring strategy, and human-oversight requirements.

### 9.6 Framework mapping

Table 11 uses CLAIM for transparent reporting of medical-imaging AI studies (93) and TRIPOD+AI when a study develops or evaluates a clinical prediction model (92). Use the FDA/Health Canada/MHRA and IMDRF GMLP principles to guide lifecycle practices for machine-learning-enabled medical devices (90, 91). Assess EU AI Act obligations separately according to jurisdiction, intended purpose, risk classification, and interaction with applicable medical-device law (94). Reporting-guideline adherence or GMLP alignment does not itself establish regulatory authorization.

**Table 11:** Regulatory and reporting framework mapping for medical VLM research and translation.

Framework/source	Jurisdiction or status	Primary purpose	Relevance to medical VLMs
FDA/Health Canada/MHRA GMLP (2021); IMDRF GMLP (2025) (90, 91)	Regulatory lifecycle principles; international harmonization	Safe, effective, high-quality ML-enabled medical-device development across the total product lifecycle	Data representativeness, independent testing, human-AI team performance, transparency, monitoring, and change management when the VLM has a medical-device intended purpose
EU AI Act, Regulation (EU) 2024/1689 (94)	Binding EU law; risk-based applicability	Legal duties for AI systems, including specified high-risk systems	Assess intended purpose, operator role, risk class, fundamental-rights duties, and interaction with medical-device conformity requirements
TRIPOD+AI (92)	International reporting guideline	Transparent reporting of clinical prediction-model development and evaluation	Apply when a VLM is used to develop or evaluate a diagnostic or prognostic prediction model; not a regulatory approval standard
CLAIM (93)	Medical-imaging AI reporting guideline	Transparent and reproducible reporting of imaging-AI studies	Use for dataset, model, reference-standard, evaluation, and clinical-context reporting; not a legal authorization pathway
